# Supplementary material for: VSV-Displayed HIV-1 Envelope Identifies Broadly Neutralizing Antibodies Class-Switched to IgG and IgA
Source: Cell Host Microbe. 2020 Jun 10;27(6):963–975.e5. doi: 10.1016/j.chom.2020.03.024 (PMC7294236; doi:10.1016/j.chom.2020.03.024)
Supplement: Document S1. Figures S1–S3 and Tables S1–S4 [file mmc1.pdf]

Cell Host & Microbe, Volume 27

Supplemental Information

VSV-Displayed HIV-1 Envelope Identifies Broadly Neutralizing Antibodies Class-Switched to IgG and IgA

Manxue Jia, Rachel A. Liberatore, Yicheng Guo, Kun-Wei Chan, Ruimin Pan, Hong Lu, Eric Waltari, Eva Mittler, Kartik Chandran, Andrés Finzi, Daniel E. Kaufmann, Michael S. Seaman, David D. Ho, Lawrence Shapiro, Zizhang Sheng, Xiang-Peng Kong, Paul D. Bieniasz, and Xueling Wu

Table S1. Neutralization IC_{50} (µg/mL) of bNAbs (expressed as IgG1 or otherwise indicated) against 120 HIV-1 Env pseudoviruses. Related to Figure 1.

Virus	Clade	M4008		M1214		M1214	
		N1	N1.3 dIgA2	N1	N1.2	N2	N2.2
MS208.A1	A	>50	>50	40.2	0.090	>50	>50
Q23.17	A	0.170	0.261	1.3	0.182	>50	>50
Q461.e2	A	23.0	>50	3.0	11.4	>50	>50
Q769.d22	A	>50	>50	>50	>50	>50	>50
Q259.d2.17	A	10.8	>50	>50	>50	>50	>50
Q842.d12	A	0.082	0.414	>50	>50	0.916	>50
0260.v5.c36	A	>50	>50	>50	>50	>50	26.2
191955_A11	A (T/F)	4.0	35.1	0.205	27.1	>50	>50
191084 B7-19	A (T/F)	>50	>50	0.030	0.023	0.020	0.107
9004SS_A3_4	A (T/F)	2.0	>50	0.738	1.4	0.008	35.3
BG505	A (T/F)	0.112	0.865	>50	0.238	>50	0.518
6535.3	B	1.2	36.2	>50	>50	>50	>50
QH0692.42	B	0.203	0.096	0.045	0.035	0.111	>50
SC422661.8	B	>50	>50	0.016	0.070	0.018	0.026
PVO.4	B	0.160	0.132	0.192	2.9	0.019	0.420
TRO.11	B	0.124	7.4	>50	>50	0.027	0.017
AC10.0.29	B	1.1	>50	0.067	0.063	>50	1.3
RHPA4259.7	B	0.815	>50	0.004	0.011	0.364	>50
THRO4156.18	B	>50	>50	>50	21.1	>50	>50
REJO4541.67	B	0.133	0.152	0.015	0.145	>50	48.1
TRJO4551.58	B	>50	>50	10.2	0.025	>50	>50
WITO4160.33	B	0.197	>50	0.093	0.263	0.083	>50
CAAN5342.A2	B	>50	>50	0.024	44.3	>50	>50
JR-FL	B	0.004	0.019	0.009	0.096	0.019	0.027
Yu2	B	0.163	0.186	0.026	0.109	0.114	0.035
BL01	B	>50	>50	>50	>50	4.0	0.070
WEAU_d15_410_5017	B (T/F)	>50	>50	>50	0.336	0.059	0.041
1006_11_C3_1601	B (T/F)	0.109	>50	0.016	0.027	0.090	>50
1054_07_TC4_1499	B (T/F)	>50	>50	0.046	0.374	>50	>50
1056_10_TA11_1826	B (T/F)	0.177	0.070	0.271	1.2	>50	>50
1012_11_TC21_3257	B (T/F)	0.055	0.612	0.063	0.990	0.058	0.009
6240_08_TA5_4622	B (T/F)	13.0	>50	0.986	29.3	0.030	0.020
6244_13_B5_4576	B (T/F)	1.0	1.6	0.724	0.815	>50	0.060
62357_14_D3_4589	B (T/F)	8.6	>50	0.036	0.404	0.915	1.1
SC05_8C11_2344	B (T/F)	>50	>50	6.4	1.2	0.014	0.012
AD17	B (T/F)	0.295	0.342	0.039	0.532	0.181	>50
Du156.12	C	>50	>50	>50	>50	>50	>50
Du172.17	C	>50	>50	>50	0.468	>50	>50
Du422.1	C	>50	>50	>50	>50	>50	>50
ZM197M.PB7	C	2.6	9.2	>50	>50	>50	18.1
ZM214M.PL15	C	>50	>50	16.0	20.2	>50	>50
ZM233M.PB6	C	>50	43.5	14.2	>50	>50	>50
ZM249M.PL1	C	>50	>50	>50	>50	>50	>50
ZM53M.PB12	C	>50	>50	24.3	0.285	>50	>50
ZM109F.PB4	C	0.046	0.741	>50	>50	>50	>50
ZM135M.PL10a	C	>50	>50	>50	>50	>50	>50
CAP45.2.00.G3	C	>50	>50	>50	>50	>50	>50
CAP210.2.00.E8	C	>50	>50	>50	>50	>50	>50
HIV-001428-2.42	C	0.762	0.578	0.457	0.104	>50	>50
HIV-0013095-2.11	C	>50	>50	>50	>50	>50	>50
HIV-16055-2.3	C	>50	>50	>50	>50	>50	>50
HIV-16845-2.22	C	>50	>50	>50	>50	>50	>50
Ce1086_B2	C (T/F)	13.1	>50	2.5	11.7	>50	>50
Ce0393_C3	C (T/F)	>50	>50	>50	>50	>50	>50
Ce1176_A3	C (T/F)	>50	>50	0.463	32.3	>50	>50
Ce2010_F5	C (T/F)	9.1	>50	0.601	0.081	>50	>50
Ce0682_E4	C (T/F)	>50	27.4	>50	>50	>50	>50
Ce1172_H1	C (T/F)	17.8	12.4	>50	>50	>50	>50
Ce2060_G9	C (T/F)	>50	>50	0.019	0.084	>50	>50
Ce703010054_2A2	C (T/F)	>50	>50	>50	14.8	>50	>50
BF1266.431a	C (T/F)	1.1	1.5	>50	>50	>50	>50
246F C1G	C (T/F)	0.028	1.5	>50	0.529	>50	>50
249M B10	C (T/F)	>50	>50	>50	7.2	>50	>50
ZM247v1(Rev-)	C (T/F)	>50	>50	0.100	3.6	>50	>50
7030102001E5(Rev-)	C (T/F)	>50	>50	11.3	2.6	0.008	0.023
1394C9G1(Rev-)	C (T/F)	>50	>50	>50	>50	>50	>50
Ce704809221_1B3	C (T/F)	2.5	>50	>50	>50	>50	>50

Table S1. Neutralization IC_{50} (μg/mL) of bNAbs (expressed as IgG1 or otherwise indicated) against 120 HIV-1 Env pseudoviruses. Related to Figure 1. *Continued.*

Virus	Clade	M4008		M1214		M1214	
		N1	N1.3 dIgA2	N1	N1.2	N2	N2.2
CNE19	BC	0.269	0.156	0.008	0.024	>50	>50
CNE20	BC	>50	>50	3.3	>50	>50	>50
CNE21	BC	>50	>50	0.038	47.5	>50	>50
CNE17	BC	>50	13.1	0.075	0.238	>50	>50
CNE30	BC	>50	>50	0.963	2.8	>50	>50
CNE52	BC	>50	>50	>50	>50	>50	>50
CNE53	BC	>50	>50	22.8	3.6	>50	>50
CNE58	BC	22.2	0.284	>50	45.3	>50	>50
620345.c01	CRF01_AE	>50	>50	>50	>50	>50	>50
C1080.c03	CRF01_AE	>50	>50	>50	>50	>50	>50
R2184.c04	CRF01_AE	>50	>50	0.008	0.099	>50	>50
R1166.c01	CRF01_AE	>50	>50	7.9	>50	>50	>50
R3265.c06	CRF01_AE	>50	>50	0.230	>50	>50	>50
C2101.c01	CRF01_AE	>50	>50	0.006	0.123	>50	>50
C3347.c11	CRF01_AE	>50	>50	0.517	>50	>50	>50
C4118.c09	CRF01_AE	>50	>50	6.9	30.0	>50	>50
CNE5	CRF01_AE	>50	>50	0.973	0.255	>50	>50
BJOX009000.02.4	CRF01_AE	>50	>50	13.6	3.3	>50	>50
BJOX015000.11.5	CRF01_AE (T/F)	>50	>50	0.056	0.323	>50	>50
BJOX010000.06.2	CRF01_AE (T/F)	>50	>50	0.023	0.072	>50	>50
BJOX025000.01.1	CRF01_AE (T/F)	>50	>50	0.008	0.098	>50	>50
BJOX028000.10.3	CRF01_AE (T/F)	>50	>50	0.040	>50	>50	>50
T257-31	CRF02_AG	>50	>50	4.6	>50	>50	>50
928-28	CRF02_AG	>50	>50	>50	>50	>50	>50
263-8	CRF02_AG	>50	>50	>50	>50	0.770	>50
T250-4	CRF02_AG	>50	>50	>50	>50	0.042	>50
T251-18	CRF02_AG	>50	>50	>50	>50	>50	>50
T278-50	CRF02_AG	>50	>50	>50	>50	11.1	>50
T255-34	CRF02_AG	>50	>50	>50	>50	>50	>50
211-9	CRF02_AG	>50	>50	>50	>50	0.057	0.017
235-47	CRF02_AG	>50	>50	0.734	2.6	16.4	>50
X1193_c1	G	>50	>50	0.018	0.026	>50	>50
P0402_c2_11	G	12.7	>50	>50	>50	>50	>50
X1254_c3	G	0.926	>50	0.046	11.4	>50	>50
X2088_c9	G	>50	>50	>50	>50	>50	>50
X2131_C1_B5	G	>50	30.9	>50	>50	>50	>50
P1981_C5_3	G	>50	>50	1.1	17.2	>50	>50
X1632_S2_B10	G	0.077	>50	0.044	0.822	>50	>50
3016.v5.c45	D	>50	>50	>50	20.0	>50	>50
A07412M1.vrc12	D	>50	>50	0.115	1.6	>50	>50
231965.c01	D	>50	>50	2.3	0.153	0.054	0.768
231966.c02	D	>50	>50	0.027	0.068	>50	>50
3817.v2.c59	CD	>50	>50	13.4	>50	0.290	0.181
6480.v4.c25	CD	1.5	>50	0.067	0.178	0.130	0.068
6952.v1.c20	CD	>50	>50	>50	>50	>50	>50
6811.v7.c18	CD	0.896	0.528	0.007	0.120	>50	>50
89-F1_2_25	CD	>50	>50	>50	>50	>50	>50
3301.v1.c24	AC	45.3	>50	>50	0.391	>50	>50
6041.v3.c23	AC	0.261	0.424	>50	>50	>50	>50
6540.v4.c1	AC	0.860	6.7	>50	>50	36.2	>50
6545.v4.c1	AC	0.730	12.7	0.043	>50	6.7	>50
0815.v3.c3	ACD	0.486	0.377	>50	>50	0.055	>50
3103.v3.c10	ACD	>50	33.7	10.0	0.573	>50	>50
**MAb breadth (%)**	**N=120**	**45 (38%)**	**33 (28%)**	**68 (57%)**	**69 (57%)**	**31 (26%)**	**24 (20%)**
**Clone breadth (%)**		**50 (42%)**		**78 (65%)**		**37 (31%)**	
**MAb breadth (%) IC_{50} < 1 μg/mL**		**26 (22%)**	**18 (15%)**	**48 (40%)**	**42 (35%)**	**26 (22%)**	**18 (15%)**
**Clone breadth (%) IC_{50} < 1 μg/mL**		**27 (23%)**		**59 (49%)**		**29 (24%)**	
**MAb geometric mean (μg/mL)**		**0.755**	**1.408**	**0.255**	**0.702**	**0.154**	**0.199**
**Clone geometric mean**		**0.766**		**0.188**		**0.159**	

IC_{50} color code

< 1	1.0 - 50	>50 (μg/mL)

Table S2. Key contacts of the M1214_N1 epitope on gp120 and their levels of conservation among 5,164 global Env sequences (version year 2016) retrieved from the Los Alamos HIV sequence database (http://www.hiv.lanl.gov/). Related to Figure 5.

Gp120 residues	Gp120 regions	Buried surface (Å^2)#	Level of conservation*
Q183	V2	14.9	0.572
D185	V2	34.1	0.365
I194	V2	15.2	0.806
N195	V2	11.3	0.670
N280	C2, loop D	23.0	0.953
S365	C3, CD4 binding loop	18.7	0.787
G366	C3, CD4 binding loop	10.4	0.990
D368	C3, CD4 binding loop	25.9	0.992
L369	C3, CD4 binding loop	15.1	0.639
E370	C3, CD4 binding loop	32.8	0.987
Y384	C3, CD4 binding site	25.7	0.968
K421	C4	15.9	0.892
I424	C4	16.5	0.797
N425	C4	39.1	0.871
D457	C4	22.0	0.981
R469	V5	23.0	0.981
Geometric Mean			0.804

#The buried surface areas were calculated using ICM software package (https://www.molsoft.com/), and only residues with surface contact areas > 10 Å^2 are listed.
*Level of conservation was calculated using an entropy scoring method implemented in the R package bio3d.

Table S3. M1214_N1 Fab crystallographic data collection and refinement statistics. Related to Figure 5.

Protein	M1214_N1 Fab
**PDB ID**	6VU2
**Data collection***	
Space group	P21
Cell dimensions	
a, *b*, *c* (Å)	50.7, 66.9, 70.2
α, β, γ (°)	90.0, 108.7, 90.0
Resolution (Å)	27.4-2.2 (2.27-2.19)
R_{sym} or R_{merge}	5.9 (40.7)
$I/\delta I$	10.6 (1.9)
$CC_{1/2}$	0.996 (0.933)
Completeness (%)	95.2 (72.0)
Redundancy	2.4 (1.8)
**Refinement**	
Resolution (Å)	29.4-2.2
No. reflections	37,801
R_{work}/R_{free}	17.7/21.6
No. atoms	
Protein	3258
Water	187
Wilson B-factor	37.0
r.m.s. deviations	
Bond lengths (Å)	0.007
Bond angles (º)	0.87
Ramachandran statistics	
Favored (%)	97.2
Outliers (%)	0.47

*Values in parenthesis denote highest resolution shell.

Table S4. Cryo-EM data collection, processing and refinement statistics. Related to Figure 5.

**M1214_N1 Fab/CH505 chimeric SOSIP.664 (EMD-21456, PDB 6VY2)**	
**Data collection and processing**	
Microscope	FEI Talos Arctica
Camera	Gatan K2 Summit
Magnification	36,000
Voltage (kV)	200
Electron exposure ($e^{-}/Å^2$)	80
Defocus range (μm)	-1.5 to -3.5
Pixel size (Å)	1.16
Initial particle (no.)	209,276
Final particle (no.)	94,839
Symmetry imposed	C3
Map resolution (masked, Å)	4.86
FSC threshold	0.143
Map sharpening *B* factor ($Å^2$)	-201.5
**Refinement**	
Initial models used (PDB ID)	6UDA, 6VU2
Model composition	
Non-hydrogen atoms (no.)	21429
Protein residues (no.)	2442
Sugar residues (no.)	180
RMSD	
Bond lengths (Å)	0.006
Bond angles (°)	0.905
Validation	
Clashscore	18.0
Poor rotamers (%)	5.0
Ramachandran statistics (%)	
Favored	93.8
Allowed	5.7
Outliers	0.5

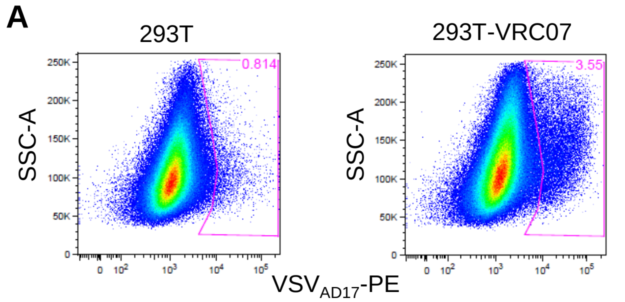

B

Virus (clade)	Plasma Neutralization ID_{50} (reciprocal dilution)	
	M4008	M1214
Q23.17 (A)	248	2,488
Q842.d12 (A)	2,080	1,440
BG505 (A)	124	994
AD17 (B)	410	256
JR-FL (B)	2,996	8,137
Yu2 (B)	429	682
AC10.29 (B)	338	305
BL01 (B)	86	120
Du156.12 (C)	176	242
ZM109.4 (C)	111	<50

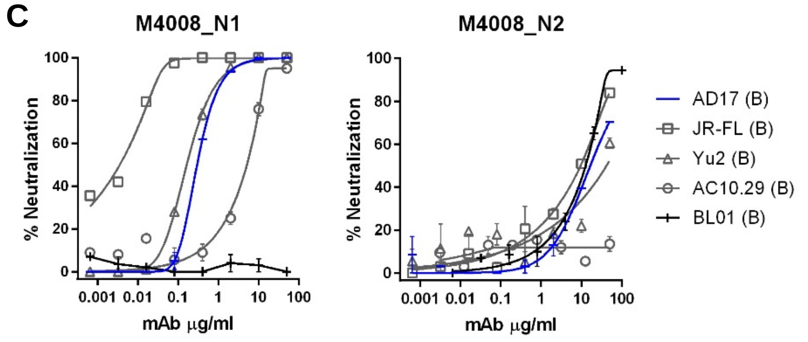

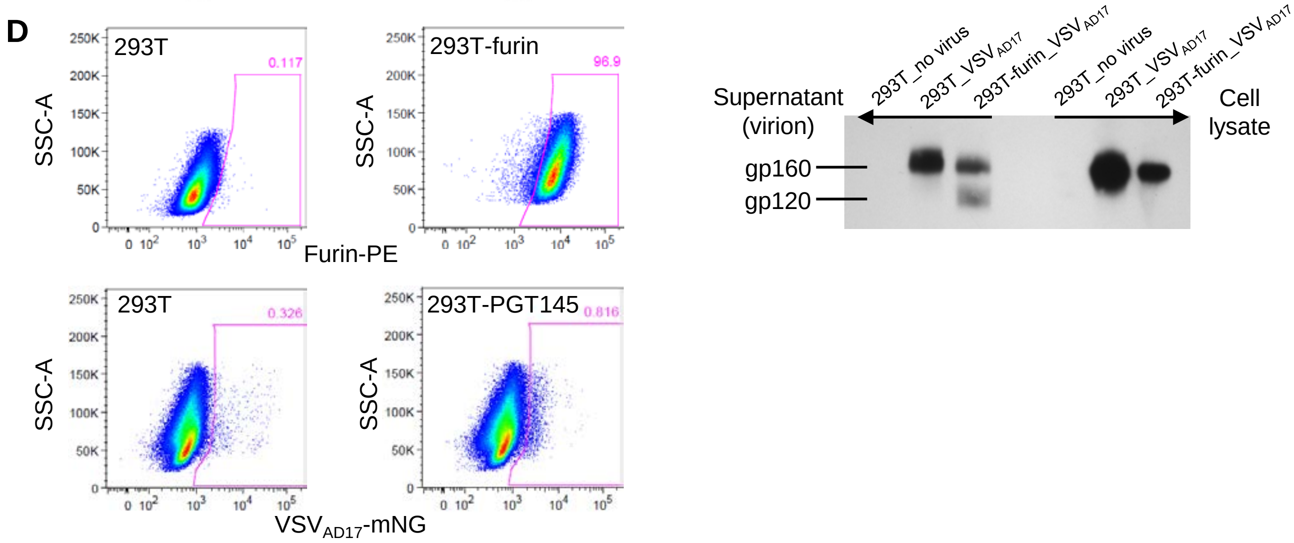

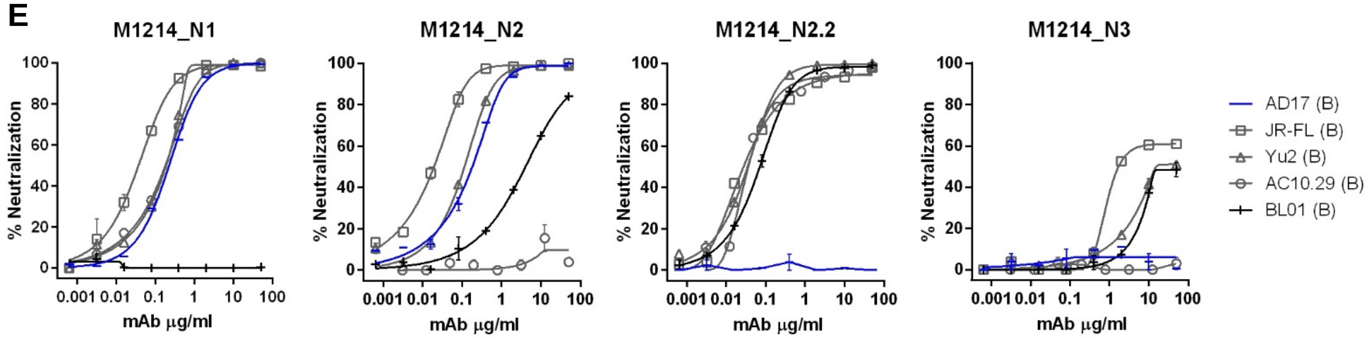

Figure S1. Evaluation of VSV_{AD17} as B cell probe for HIV-1 bNAb isolation. Related to Figure 1.
(**A**) Flow cytometry analysis of VSV_{AD17}-PE binding to 293T cells and to 293T cells expressing membrane-bound VRC07 (293T-VRC07). SSC-A: side-scatter area.
(**B**) Neutralization ID_{50}, shown as plasma reciprocal dilution, of two donors from the Montreal HIV-1 infection cohort, against 10 HIV-1 Env strains as indicated, with the probe strain AD17 in blue.
(**C**) Neutralization screening of bNAbs from donor M4008 against five clade B viruses as indicated.
(**D**) Flow cytometry analysis of furin expression in 293T cells with and without stable transduction of the human furin gene; Western blot with HIVIG showing the uncleaved gp160 and cleaved gp120 in virion and in cell lysate from 293T versus 293T-furin; flow cytometry analysis of VSV_{AD17}-mNG staining of 293T cells and 293T cells expressing membrane-bound PGT145 (293T-PGT145).
(**E**) Neutralization screening of bNAbs from donor M1214 against five clade B viruses as indicated, with the probe strain AD17 in blue.

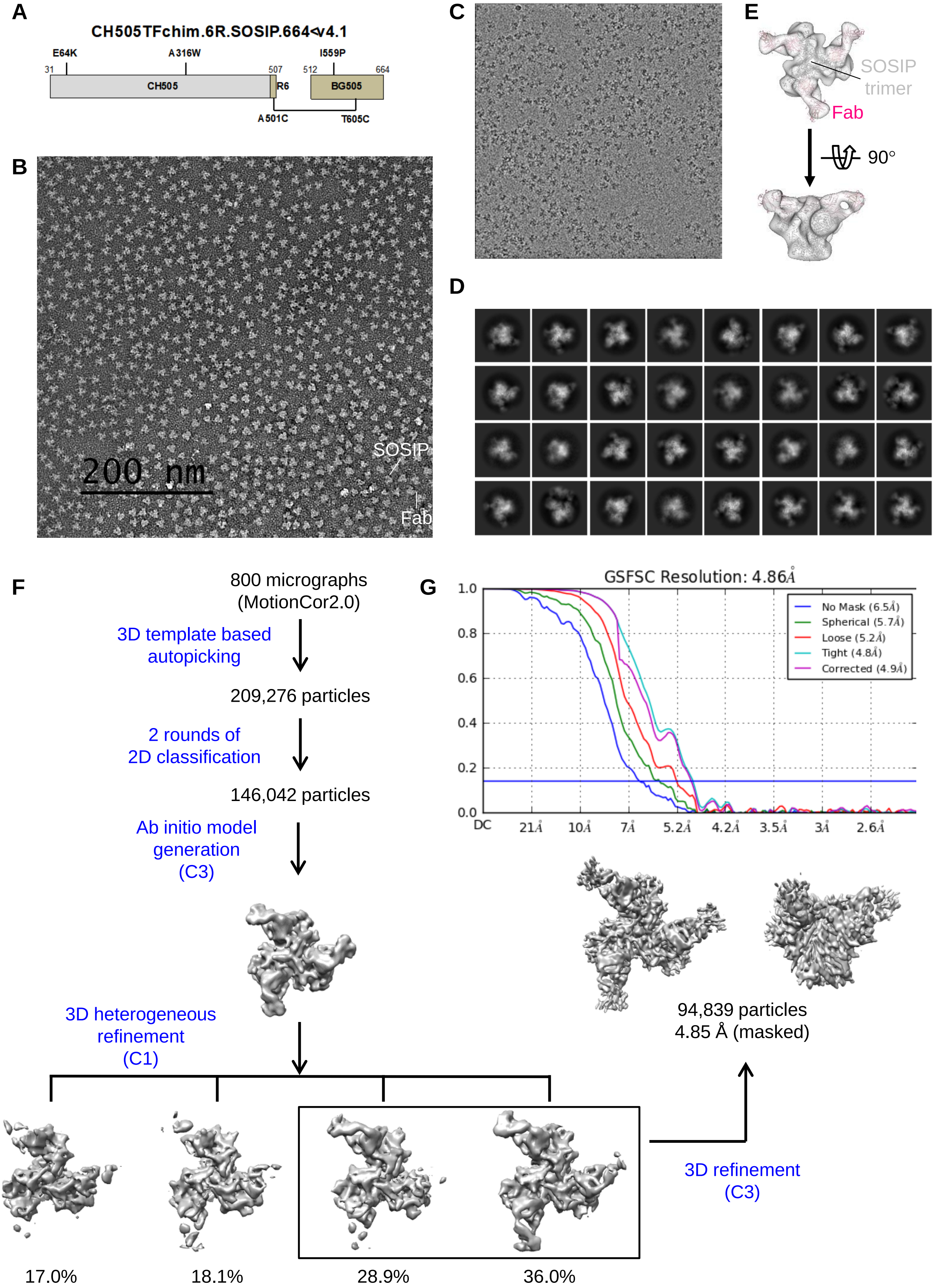
A
CH505TFchim.6R.SOSIP.664v4.1
E64K
A316W
I559P
31
507
512
664
CH505
R6
BG505
A501C
T605C
B
200 nm
SOSIP
Fab
C
D
E
SOSIP trimer
Fab
90°
F
800 micrographs (MotionCor2.0)
3D template based autopicking
209,276 particles
2 rounds of 2D classification
146,042 particles
Ab initio model generation (C3)
3D heterogeneous refinement (C1)
17.0%
18.1%
28.9%
36.0%
3D refinement (C3)
94,839 particles
4.85 Å (masked)
G
GSFSC Resolution: 4.86Å
No Mask (6.5Å)
Spherical (5.7Å)
Loose (5.2Å)
Tight (4.8Å)
Corrected (4.9Å)
1.0
0.8
0.6
0.4
0.2
0.0
DC
21Å
10Å
7Å
5.2Å
4.2Å
3.5Å
3Å
2.6Å

Figure S2. Cryo-EM sample preparation, data collection and processing. Related to Figure 5.
(A) Schematic construct of the chimeric CH505 SOSIP, in which the sequence of gp41 along with the C-terminal portion of gp120 was replaced by that of BG505.
(**B**) A representative micrograph of the negative staining of M1214_N1 Fab/CH505 SOSIP complex, showing the homogeneity of the complex sample, in which more than 90% of the SOSIP trimer was bound with three Fabs (inset) estimated by 2D class averaging.
(**C**) A representative cryo-EM micrograph of M1214_N1 Fab/CH505 SOSIP complex.
(**D**) 2D averaging of cryo-EM particle images shows the structural features for both SOSIP trimer and Fab.
(**E**) 3D initial model of the M1214_N1 Fab/CH505 SOSIP complex, fitted with a CH505 SOSIP trimer (PDB 6UDA) and three crystallographic M1214_N1 Fabs (PDB 6VU2).
(**F**) Workflow of data processing performed using cryoSPAC.
(**G**) FSC curve for 3D reconstruction of the cryo-EM map. The average resolution is estimated to be 4.86 Å on the basis of the FSC value of 0.143.

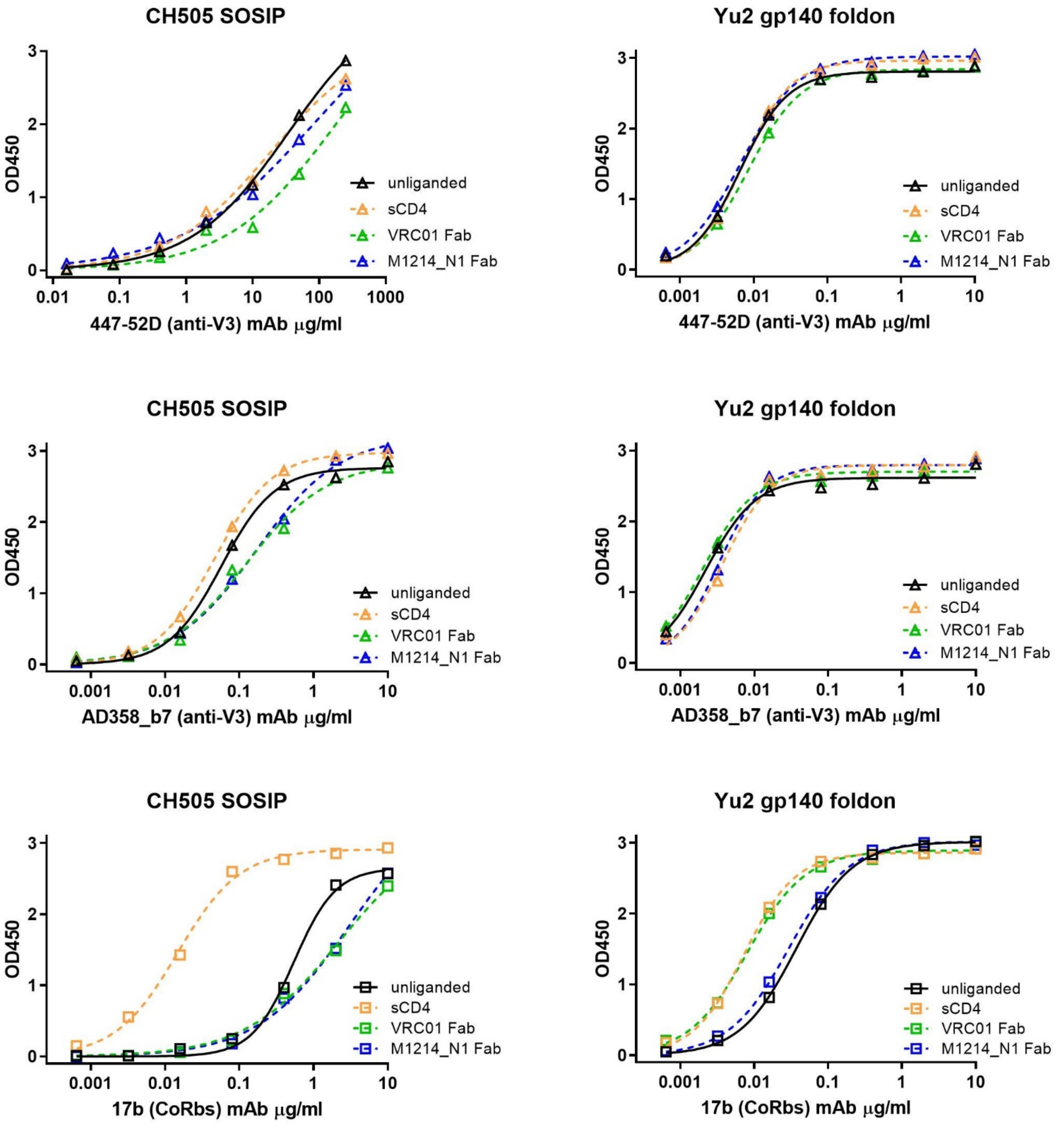

Figure S3. Assessment of the conformational change of CH505 SOSIP upon M1214_N1 Fab binding. Related to Figure 5.

The conformational change of CH505 SOSIP (left) upon 2-domain sCD4, VRC01 Fab, and M1214_N1 Fab binding was each assessed by ELISA with the anti-V3 crown mAbs 447-52D (top) and AD358_b7 (middle), as well as the coreceptor-binding site (CoRbs) mAb 17b (bottom), all of which preferentially recognize the CD4-induced “open” Env conformation. Yu2 gp140 foldon (right) was included as a control for gp140 that adopts an “open” Env conformation.
