# Supplementary material for: VSV-Displayed HIV-1 Envelope Identifies Broadly Neutralizing Antibodies Class-Switched to IgG and IgA
Source: Cell Host Microbe. 2020 Jun 10;27(6):963–975.e5. doi: 10.1016/j.chom.2020.03.024 (PMC7294236; doi:10.1016/j.chom.2020.03.024)
Supplement: Document S2. Article plus Supplemental Information [file mmc2.pdf]

Article

Cell Host & Microbe

VSV-Displayed HIV-1 Envelope Identifies Broadly Neutralizing Antibodies Class-Switched to IgG and IgA

Graphical Abstract

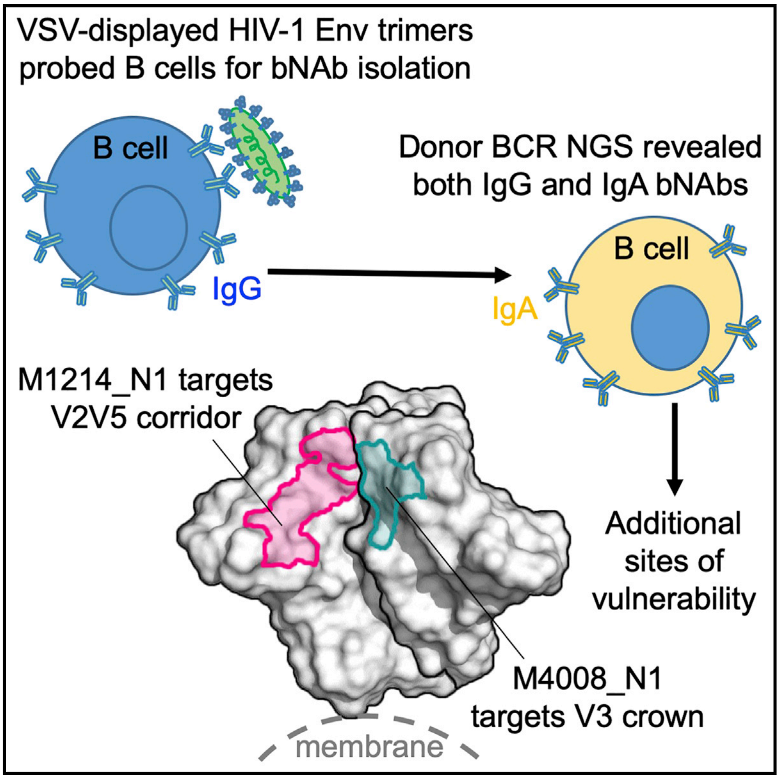

Highlights

- VSV-displayed HIV-1 envelope trimers identified five HIV-1 bNAbs
- BCR repertoires identified two bNAb lineages class-switched to both IgG and IgA
- The V3 crown-targeting bNAb M4008_N1 conferred tier 2 virus neutralization
- Cryo-EM structure of bNAb M1214_N1 with CH505 SOSIP defined a V2V5 corridor epitope

Authors

Manxue Jia, Rachel A. Liberatore, Yicheng Guo, ..., Xiang-Peng Kong, Paul D. Bieniasz, Xueling Wu

Correspondence

xw2702@cumc.columbia.edu

In Brief

Jia et al. applied a VSV-based probe that displays membrane-embedded HIV-1 envelope trimers to isolate HIV-1 bNAbs and identified two bNAb lineages that class-switched to both IgG and IgA. These bNAbs target the V3 crown and V2V5 corridor, revealing distinct sites of vulnerability on the HIV-1 envelope.

Jia et al., 2020, Cell Host & Microbe *27*, 963–975
June 10, 2020 © 2020 The Author(s). Published by Elsevier Inc.
https://doi.org/10.1016/j.chom.2020.03.024

CellPress

Cell Host & Microbe

CellPress
OPEN ACCESS

Article

VSV-Displayed HIV-1 Envelope Identifies Broadly Neutralizing Antibodies Class-Switched to IgG and IgA

Manxue Jia,[1,12] Rachel A. Liberatore,[2,3,12] Yicheng Guo,[4,12] Kun-Wei Chan,[5,12] Ruimin Pan,[5] Hong Lu,[1] Eric Waltari,[1] Eva Mittler,[6] Kartik Chandran,[6] Andrés Finzi,[7] Daniel E. Kaufmann,[7,8] Michael S. Seaman,[9] David D. Ho,[1] Lawrence Shapiro,[4,10,11] Zizhang Sheng,[4] Xiang-Peng Kong,[5] Paul D. Bieniasz,[2,3] and Xueling Wu[1,13,*]

[1]Aaron Diamond AIDS Research Center, Affiliate of The Rockefeller University, New York, NY 10016, USA
[2]Laboratory of Retrovirology, The Rockefeller University, New York, NY 10065, USA
[3]Howard Hughes Medical Institute, New York, NY 10016, USA
[4]Zuckerman Mind Brain Behavior Institute, Columbia University, New York, NY 10027, USA
[5]Department of Biochemistry and Molecular Pharmacology, New York University School of Medicine, New York, NY 10016, USA
[6]Department of Microbiology and Immunology, Albert Einstein College of Medicine, Bronx, NY 10461, USA
[7]Centre de Recherche du CHUM and Université de Montréal, Montreal, QC H2X 0A9, Canada
[8]Center for HIV-1/AIDS Vaccine Immunology and Immunogen Discovery (CHAVI-ID), La Jolla, CA 92037, USA
[9]Beth Israel Deaconess Medical Center, Harvard Medical School, Boston, MA 02115, USA
[10]Department of Biochemistry and Molecular Biophysics, Columbia University, New York, NY 10032, USA
[11]Vaccine Research Center, National Institute of Allergy and Infectious Diseases, National Institutes of Health, Bethesda, MD 20892, USA
[12]These authors contributed equally
[13]Lead Contact
*Correspondence: xw2702@cumc.columbia.edu
https://doi.org/10.1016/j.chom.2020.03.024

SUMMARY

The HIV-1 envelope (Env) undergoes conformational changes during infection. Broadly neutralizing antibodies (bNAbs) are typically isolated by using soluble Env trimers, which do not capture all Env states. To address these limitations, we devised a vesicular stomatitis virus (VSV)-based probe to display membrane-embedded Env trimers and isolated five bNAbs from two chronically infected donors, M4008 and M1214. Donor B cell receptor (BCR) repertoires identified two bNAb lineages, M4008_N1 and M1214_N1, that class-switched to immunoglobulin G (IgG) and IgA. Variants of these bNAbs reconstituted as IgA demonstrated broadly neutralizing activity, and the IgA fraction of M1214 plasma conferred neutralization. M4008_N1 epitope mapping revealed a glycan-independent V3 epitope conferring tier 2 virus neutralization. A 4.86-Å-resolution cryogenic electron microscopy (cryo-EM) structure of M1214_N1 complexed with CH505 SOSIP revealed another elongated epitope, the V2V5 corridor, extending from V2 to V5. Overall, the VSV_{ENV} probe identified bNAb lineages with neutralizing IgG and IgA members targeting distinct sites of HIV-1 Env vulnerability.

INTRODUCTION

HIV-1 broadly neutralizing antibodies (bNAbs), when passively administered, demonstrated viral suppression in patients (Caskey et al., 2015; Caskey et al., 2019; Lynch et al., 2015) and protection in simian/human immunodeficiency virus (SHIV) macaque challenge models (Pegu et al., 2019). To date, the isolated HIV-1 bNAbs fall into seven categories defining seven major conserved sites of vulnerability on the HIV-1 envelope (Env)—namely the first and second variable (V1V2) apex (McLellan et al., 2011; Walker et al., 2009), V3 glycan supersite (Pejchal et al., 2011; Walker et al., 2011), CD4-binding site (CD4bs) (Scheid et al., 2011; Wu et al., 2010; Zhou et al., 2010), silent face (Schoofs et al., 2019; Zhou et al., 2018), subunit interface (Falkowska et al., 2014; Huang et al., 2014), fusion peptide (Kong et al., 2016), and membrane proximal external region (MPER) (Huang et al., 2012; Williams et al., 2017). The majority of these bNAbs have been discovered by using soluble probes produced with Envglycoprotein (gp120) monomers or gp140 stabilized trimers. Because the HIV-1 Env adopts multiple trimeric conformations (Lu et al., 2019; Ma et al., 2018), current soluble Env probes might not fully present them, and the identification of HIV-1 bNAbs remains challenging.

All of the HIV-1 bNAbs isolated to date are immunoglobulin G (IgG), the dominant antibody class in peripheral blood. However, HIV-1 spreads mainly through mucosal exposures. Dominant in mucosal secretions, IgA has long been the class of bNAbs desired at the portal of entrance to block infection (Kulkarni and Ruprecht, 2017). Unlike IgG, for which the class-switching from the IgM naive B cell receptor (BCR) occurs in the germinal centers of lymph nodes, the IgA response is predominantly elicited in the Peyer's patches of gut mucosa that favors the IgM naive BCR class-switching to IgA (Macpherson et al., 2008). The IgA response to HIV-1 might be impaired by the accelerated

This is an open access article under the CC BY license (http://creativecommons.org/licenses/by/4.0/).

loss of $CD4^+$ T cells at gut mucosa (Schneider et al., 1996); nonetheless, HIV-1-specific IgA has been documented and has a role that has been controversial (Lopez et al., 2018; Zhou and Ruprecht, 2014). On one hand, serum and mucosal IgA from infected individuals has been shown to inhibit HIV-1 (Burnett et al., 1994; Wu et al., 2003), although monoclonal IgA with neutralizing ability has not been isolated. On the other hand, serum IgA from some HIV-1-infected individuals has been reported to exhibit antibody-dependent enhancement of viral infection (Kozlowski et al., 1995), and the plasma IgA response to HIV-1 Env has been associated with increased risk of HIV-1 acquisition in the RV144 vaccine trial (Haynes et al., 2012). To address the question of whether or not IgA bNAbs are produced during HIV-1 infection and to identify additional bNAbs, we used vesicular stomatitis virus (VSV) to display functional membrane-embedded HIV-1 Env trimers (Liberatore et al., 2019) on the surface of viral particles. This approach enabled us to probe Env-specific B cells, isolate monoclonal bNAbs from HIV-1 infected individuals, and annotate the bNAb lineage members, including IgA, from the donor BCR repertoires.

RESULTS

VSV-Based Env Probes Identified HIV-1 bNAbs

Compared with HIV-1 virions or virus-like particles (Cale et al., 2017), the VSV particles presented two advantages: first, VSV particles display more Env spikes on the surface—the number of Env spikes on HIV-1 is estimated to be about a dozen (Klein and Bjorkman, 2010; Zhu et al., 2003; Zhu et al., 2006), whereas the number on VSV is in the hundreds (Thomas et al., 1985), thus increasing the avidity for B cell binding; and second, in the case of viral membrane rupture, the exposure of VSV matrix and capsid does not lead to cross-reactivity with memory B cells targeting the HIV-1 Gag that is irrelevant to virus neutralization. Using a molecular clone of VSV (Indiana strain), we replaced the extracellular and transmembrane domains of VSV-G with the AD17 HIV-1 Env, a clade B transmitted founder (T/F) strain (Li et al., 2010) but retained the intracellular portion of VSV-G to facilitate Env incorporation (Figure 1A). The recombinant VSV_{AD17} was rescued by transfection in 293T cells, expanded in GHOST.R5 cells, and labeled with phycoerythrin (PE). The labeled VSV_{AD17}-PE particles stained 3.55% of 293T cells expressing VRC07 (Rudicell et al., 2014) on the surface, compared with 0.8% of unmodified 293T cells (Figure S1A). We applied this probe to a clade B chronically infected subject, M4008, whose plasma neutralized all of the 10 HIV-1 Env strains tested, with a 50% inhibitory dilution (ID_{50}) against AD17 at about 1:400 (Figure S1B). From 4 million M4008 peripheral blood mononuclear cells (PBMCs), we pre-sorted 0.16 million $CD3^-CD19^+$ B cells, then stained them with VSV_{AD17}-PE and isolated 87 IgG^+PE^+ B cells that constituted ~1.5% of total IgG^+ B cells (Figure 1B). Using single-B-cell RT-PCR, we recovered two bNAbs, M4008_N1 and M4008_N2 (Figure S1C), as well as six tier-1-neutralizing or non-neutralizing monoclonal antibodies (mAbs) (Table 1).

To improve the sensitivity and specificity of the VSV_{AD17} probe, we next used a VSV backbone encoding monomeric NeonGreen (mNG)—a bright green fluorescent protein variant, fused to the VSV-P gene (Kleinfelter et al., 2015; Spence et al., 2016) (Figure 1A). We also propagated the virus in GHOST.R5 cells transfected with VSV-G, followed by a single round of replication through 293T-furin cells that overexpressed human furin to improve HIV-1 Env cleavage (Figure S1D). The resulting VSV_{AD17}-mNG stained ~0.3% of unmodified 293T, whereas it gave a 2- to 3-fold higher signal (0.8%) on 293T cells expressing surface PGT145 (Walker et al., 2011) (Figure S1D). We applied VSV_{AD17}-mNG to another clade B chronically infected subject, M1214, whose plasma neutralized 9 out of 10 HIV-1 Env strains tested, with an ID_{50} against AD17 at about 1:250 dilution (Figure S1B). From 50 million M1214 PBMCs, we pre-sorted 0.5 million $CD3^-CD19^+$ B cells, then stained with VSV_{AD17}-mNG and sorted 51 mNG^+ B cells that constituted ~0.1% of IgM^-CD27^+ memory B cells (Figure 1C). Using single-B-cell RT-PCR, we recovered four bNAbs, M1214_N1, M1214_N2, a clonal variant M1214_N2.2, and M1214_N3 (Figure S1E), and one non-neutralizing mAb, M1214_b4 (Table 1).

Of the five unique bNAbs isolated, we tested three (M4008_N1, M1214_N1, and M1214_N2), along with their distant clonal variants, for neutralization of an extended multi-clade panel of 120 Env isolates (Figure 1D and Table S1). The other two bNAbs, M4008_N2 and M1214_N3, were not tested in the extended panel because of weak and partial neutralization, respectively (Figures S1C and S1E). The M1214_N1 clone exhibited the best breadth (65%) and potency (0.19 μg/mL geometric mean 50% inhibitory concentration [IC_{50}]) against the 120 Env panel, followed by the M4008_N1 clone with 42% breadth and 0.77 μg/mL potency and the M1214_N2 clone with 31% breadth and 0.16 μg/mL potency. Both M4008_N1 and M1214_N2 exhibited a clade B preference, neutralizing 17 out of 25 (68%) clade B Envs tested. Interestingly, M4008_N2, M1214_N2, M1214_N2.2, and M1214_N3 neutralized the difficult-to-neutralize clade B strain BL01, which is resistant to all previously described gp120-directed bNAbs (Figures S1C and S1E). Of the 10 HIV-1 Env strains neutralized by donor M4008 plasma (Figure S1B), the isolated bNAbs M4008_N1 and M4008_N2 neutralized 9, the exception being Du156.12 (Figure S1C and Table S1). Similarly, of the 9 HIV-1 Env strains sensitive to donor M1214 plasma (Figure S1B), M1214_N1 and M1214_N2 neutralized 8, again the exception being Du156.12 (Table S1). Overall, the VSV_{AD17} probe efficiently identified multiple bNAb clones from each donor, and the isolated bNAb clones accounted for the majority of the donor plasma neutralizing activity.

Identification of bNAb Lineages That Class-Switched to Both IgG and IgA

From the donor PBMCs, we also amplified and sequenced the expressed BCR messenger RNA (mRNA) spanning the variable region of μ, γ, α, κ, and λ chains by using 5′ rapid amplification of cDNA ends (RACE) PCR and performed Illumina sequencing (Waltari et al., 2018). Using the isolated bNAbs as references, we produced divergence-identity plots to identify additional members of the bNAb lineages (Figure 2A). For M4008_N1 and M1214_N1, the BCR repertoire identified clonally related γ and corresponding light-chain transcripts, but not μ transcripts, as expected. Surprisingly, the BCR repertoire also identified clonally related α transcripts, indicating that these bNAb lineages class-switched to both IgG and IgA, a phenomenon unknown for HIV-1 bNAbs before. Further phylogenetic analyses of clonal members of M4008_N1 and M1214_N1 supported both direct

CellPress
OPEN ACCESS

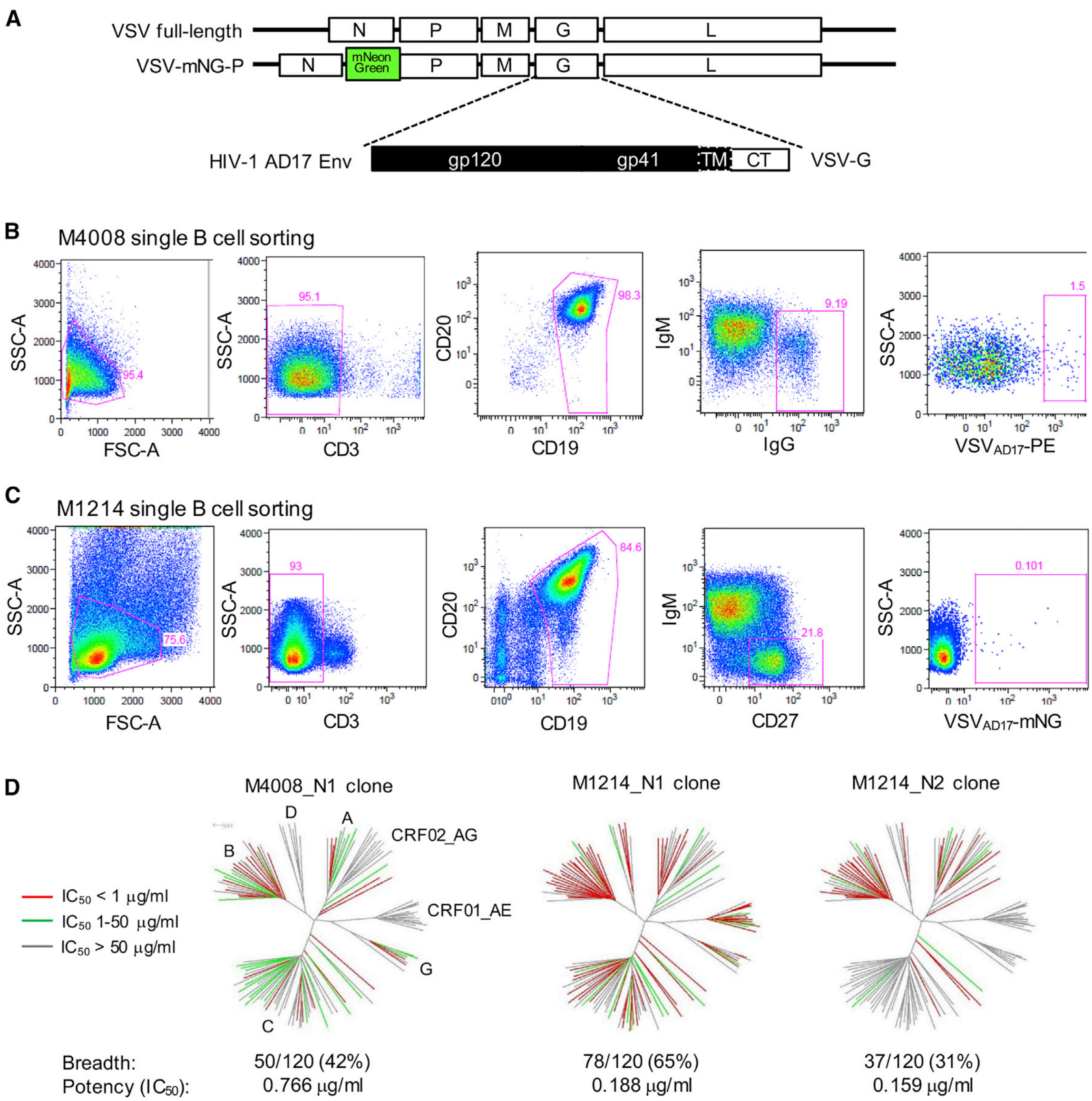

Figure 1. VSV_{AD17} as B Cell Probe for HIV-1 bNAb Isolation

(A) Schematic VSV_{AD17} constructs. Abbreviations are as follows: mNG, monomeric NeonGreen; TM, transmembrane; CT, cytoplasmic tail.

(B) Fluorescence-activated cell sorting (FACS) of pre-sorted B cells from donor M4008 for single IgG^{+} memory B cells bound to VSV_{AD17}-PE. Abbreviations are as follows: SSC-A, side-scatter area; FSC-A, forward-scatter area.

(C) FACS of pre-sorted B cells from donor M1214 for single $IgM^{-}CD27^{+}$ memory B cells bound to VSV_{AD17}-mNG.

(D) Neutralization breadth and potency of three bNAb clones from M4008 and M1214 evaluated on a total of 120 cross-clade HIV-1 Env strains. The dendrograms show the Env protein sequence distance, with clades, including circulating recombinant forms (CRFs), indicated. The neutralization potency is color-coded for the branch of each Env tested. The data under the dendrograms show the number and percent of viruses neutralized with an $IC_{50} < 50$ µg/mL and the geometric mean IC_{50} value for viruses neutralized with an $IC_{50} < 50$ µg/mL.

(primary) and indirect (secondary) class-switch mechanisms generating the observed IgA transcripts (Figure 2B). For M4008_N1.3 IgA and M1214_N1.2 IgA branches, there were closely related IgG reads consistent with a secondary class-switch from IgG to IgA. Governed by the deletional process of class-switch recombination (CSR) that splices out the constant genes between the antibody variable region and the selected downstream constant gene (Chaudhuri and Alt, 2004), switching from IgG to IgA is theoretically plausible given the genomic locations of human immunoglobulin heavy-chain constant genes (Figure 2C). Because the PCR primers used for the donor BCR next-generation sequencing (NGS) did not distinguish among IgG and IgA subclasses, we could only infer that, for the probe-identified M4008_N1 IgG2 lineage, its secondary class-switching from variant N1.3 IgG to N1.3 IgA would be IgA2, and for the probe-identified M1214_N1 IgG1 lineage, its secondary class-switching from variant N1.2 IgG to N1.2 IgA could be IgA1 or IgA2 (Figure 2C). Although supported by data from B

Table 1. Summary of the Isolated HIV-1 bNAbs and Env-Specific mAbs for Isotype, Genetic Composition, Epitope, and Neutralization Characteristics

mAb ID	Donor	Isotype	V-gene*(hypermutation)	CDR3 length(amino acid)	Epitope	Neutralization
M4008_N1	M4008	IgG2 (IgA)	VH1-69 (28%) VK1-5 (24%)	H3: 17, L3: 9	V3 loop	tier-2 (B), 38% breadth
M4008_N2	M4008	IgG3	VH4-61 (28%) VL3-21 (11%)	H3: 15, L3: 11	Env trimer or gp41	tier-2, weak potency
M4008_b3	M4008	IgG1	VH3-30 (14%) VL1-47 (15%)	H3: 20, L3: 11	gp120, CD4bs*	tier-1
M4008_b3.2	M4008	IgG1	VH3-30 (19%) VL1-47 (12%)	H3: 20, L3: 11	gp120, CD4bs	tier-1
M4008_b4	M4008	IgG1	VH1-18 (16%) VK3-20 (12%)	H3: 21, L3: 9	gp120, CD4bs	tier-1
M4008_b5	M4008	IgG1	VH4-34 (13%) VK4-1 (3%)	H3: 23, L3: 10	gp120, V3 tip	tier-1
M4008_b6	M4008	IgG1	VH1-69-2 (15%) VK1-39 (12%)	H3: 14, L3: 9	gp120, V3 tip	tier-1
M4008_b7	M4008	IgG3	VH3-30 (15%) VK2-28 (15%)	H3: 17, L3: 10	gp41	None
M1214_N1	M1214	IgG1 (IgA)	VH3-66 (35%) VL2-11 (24%)	H3: 18, L3: 11	V2V5 corridor	tier-2, 57% breadth
M1214_N2	M1214	IgG1	VH4-34 (30%) VK2-24 (17%)	H3: 11, L3: 9	V3 glycans	tier-2 (B), 26% breadth
M1214_N2.2	M1214	IgG1	VH4-34 (27%) VK2-24 (20%)	H3: 11, L3: 9	V3 glycans	tier-2 (B), 20% breadth
M1214_N3	M1214	IgG1	VH6-1 (24%) VL1-44 (20%)	H3: 19, L3: 11	V3 glycans	tier-2, partial neutralization
M1214_b4	M1214	IgG1	VH1-69 (26%) VK3-20 (20%)	H3: 15, L3: 8	gp41	None

An * indicates the mAb sequences were assigned to the closest known human germline V-genes as appropriate; Abbreviation is as follows: CD4bs, CD4-binding site.

cell cultures (Zan et al., 1998) and BCR sequencing (Horns et al., 2016; Lin et al., 2014), here the IgG to IgA switching mechanism was evident in human antibodies with known specificity and function. For the M4008_N1.4 IgA branch, however, no IgG transcript was closely clustered with IgA transcripts that would support an IgG ancestor. Thus, a primary class-switch mechanism from a common IgM ancestor (M4008_N1) to both IgG and IgA appeared more likely. Because the BCR repertoire data did not contain the IgA subclass information, the M4008_N1.4 IgA cluster could be IgA1 or IgA2 (Figure 2C). To assess the function of NGS-identified IgG and IgA lineage members, we synthesized the representative heavy- and light-chain sequences of each clonal cluster (Figure 3A) and expressed the clonal variants as monomeric IgG1, IgA1, or IgA2 or dimeric IgA2 (dIgA2). The clonal variants of M4008_N1 and M1214_N1 demonstrated various degrees of neutralizing activity (Figure 3B), and some were comparable to that of the prototype bNAbs M4008_N1 and M1214_N1 (Figures S1C and S1E). To assess the physiological relevance of the IgA bNAb members and help rule out possible sequencing or PCR artifacts, we purified the IgA fraction from M1214 plasma and found that the M1214 plasma IgA indeed neutralized the same four HIV-1 Env strains as did the M1214_N1.2 IgA1 bNAb (Figure 3B). Thus, IgA bNAbs were produced during HIV-1 infection and contributed to the overall plasma neutralizing activity. For donor M4008, however, there was insufficient plasma available to test purified IgA neutralization activity. We note that only a subset of bNAbs class-switched to both IgG and IgA. For example, all members of the M1214_N2 bNAb lineage contained only IgG but no IgA or IgM transcripts (Figure 2A). We speculate that in these donors, a local environment favoring IgA class-switch (Cerutti, 2008) might have promoted and selected certain bNAb clones, either as activated IgM or clonally expanded IgG, to further switch to IgA. The lack of IgM detection might have resulted from the relative rarity of circulating gut-derived IgM$^+$ memory B cells and plasma cells, although in principle, mucosal pentameric IgM could protect against HIV-1 (Gong et al., 2018). Indeed, mucosal surfaces are likely a more appropriate place for the detection of IgM bNAbs (Magri et al., 2017), particularly in infected individuals with a concomitant primary or secondary IgA deficiency.

Site of Vulnerability Mapped to gp120 V3 Loop

We next mapped the epitopes of the isolated bNAbs, first by comparing neutralization sensitivities of the HIV-1 strain JR-FL between the wild-type (WT) and mutant that lacked a glycan at N160, N301, or N332. Compared with that of WT JR-FL, the neutralization sensitivities of the mutant viruses were either comparable or enhanced for M4008_N1, M4008_N2, and M1214_N1; however, the mutations removing a glycan at N301 or N332 substantially reduced or abolished M1214_N2 and M1214_N3 neutralizing activity, indicating that the V3 stem glycans at N301 and/or N332 were a target for these bNAbs (Figure 4A). Competition ELISA showed that known V3-directed mAbs such as PGT128, 447-52D, 2219, and 2424 each effectively competed with M4008_N1-biotin binding to JR-FL gp120 (Figure 4B), suggesting that the epitope of M4008_N1 involved the V3 loop; meanwhile, CD4-Ig but not CD4bs-directed bNAbs also blocked M4008_N1-biotin binding. Unlike PGT128, which relies on the V3 stem glycans at N301 and N332 for gp120 binding (Pejchal et al., 2011), M4008_N1 was glycan-independent given that it bound equally well to JR-FL gp120 treated or not treated with endoglycosidase H (Endo H). Altogether, the M4008_N1 mapping data suggested a previously undefined bNAb epitope that is V3-directed, glycan-independent, and incompatible with the CD4-triggered conformation of V3. Competition ELISA performed on CH505 SOSIP indicated a different epitope for bNAb M1214_N1, which was effectively competed by both CD4-Ig and CD4bs-directed bNAbs such as VRC01, VRC-PG04, N6, and N49P7 (Figure 4B). These data indicated that the M1214_N1 epitope overlapped with the CD4bs, and that the CD4-triggered changes in SOSIP conformation (Wang et al., 2018) disrupted the M1214_N1 epitope, because

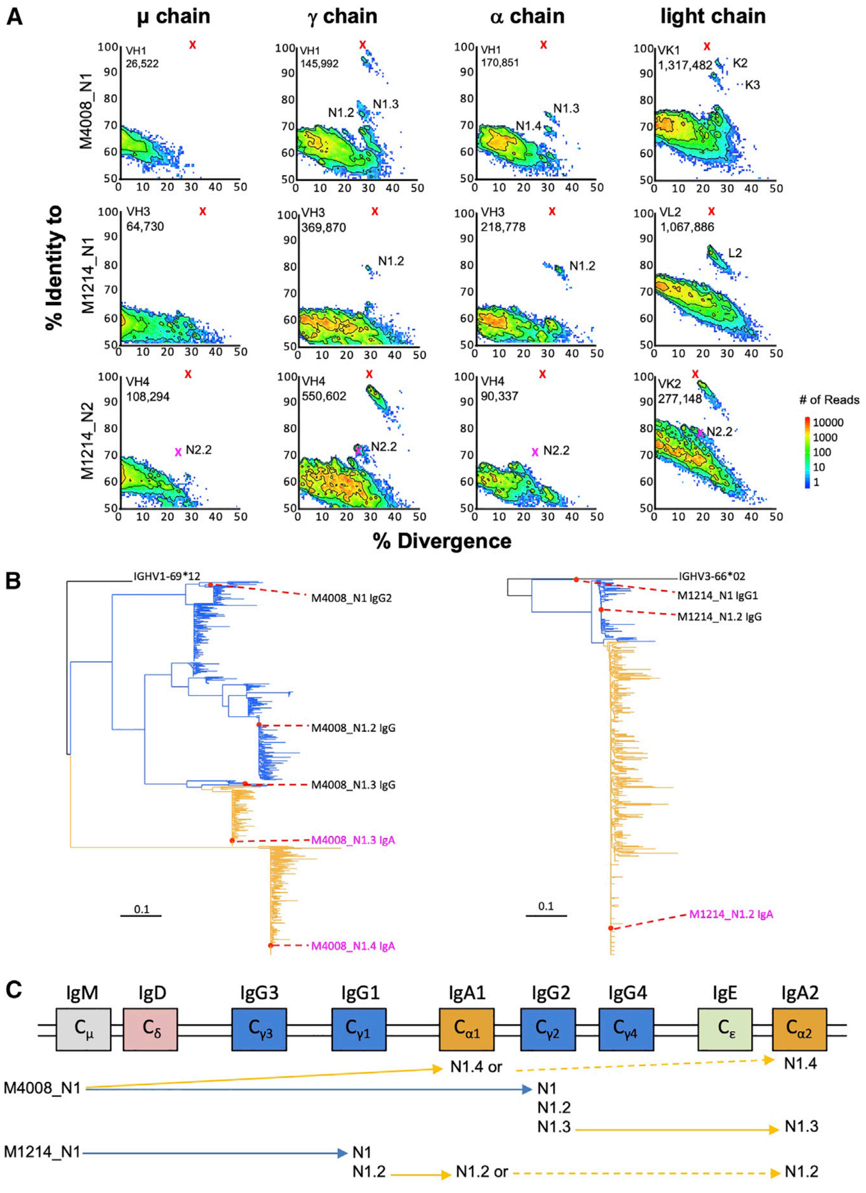

Figure 2. Identification of bNAb IgG and IgA Lineage Members by NGS of BCR Repertoires

(A) Subsets of expressed heavy-chain (μ, γ, and α) and light-chain (κ and λ) sequences are plotted as sequence identity (y axis) to each bNAb reference, as indicated, and sequence divergence (x axis) from the putative germline V-genes. The isolated bNAbs and clonal variants are marked with red and magenta Xs, respectively.

(B) Phylogenetic tree of bNAb lineage members identified by the donor BCR repertoires. Indicated are the representative sequences of each major cluster that have been synthesized and tested for neutralization function.

(C) Schematic illustrations of the order of human Ig heavy-chain constant genes and interpreted class-switching events of the two bNAb lineages, M4008_N1 and M1214_N1 (as indicated), with clonally related IgG and IgA members.

after CD4-Ig bound to CH505 SOSIP, the binding of M1214_N1-biotin was poor.

Structural Definition of the bNAb M1214_N1 Epitope

We further determined the epitope of M1214_N1 by deriving a 4.86-Å-resolution cryogenic electron microscopy (cryo-EM) structure of the antigen-binding fragment (Fab) of M1214_N1 in complex with the CH505 SOSIP trimer (Figures 5A, 5B, and S2). The structure revealed an elongated epitope of M1214_N1, extending from V2 to V5 and centered on the ridge of the CD4-binding loop (Kwong et al., 1998; Wyatt et al., 1998), thus named "V2V5 corridor" epitope (Figure 5C). The M1214_N1 epitope partially overlapped with the CD4bs at the CD4-binding loop and V5, consistent with its ability to compete with CD4-Ig and CD4bs-directed bNAbs to bind to CH505 SOSIP (Figure 4B). However, M1214_N1 approached the CD4-binding loop from the opposite (the other) side of that contacted by CD4 and CD4bs-directed bNAbs. M1214_N1 interacted with gp120 primarily through its heavy chain, which contributed almost 80% of the buried surface area of the antibody Fab. A long 18 amino acid (aa) CDR H3 loop (by Kabat definition) reached to a valley below the V1V2 β barrel (Gorman et al., 2016; Pan et al., 2015), contacting a short turn (the integrin binding site kink) (Pan et al., 2015) between the V1V2 C and C' strands (Figures 5D and 5E). The heavy chain of M1214_N1 also contacted V5 via CDR H2. The light chain of M1214_N1 contributed minor interactions with gp120, contacting residue Q183 on the C' strand of V1V2 and the glycans at N197 and N386. Interestingly, the light chain of M1214_N1 was located on the Env apex side, opposite to that of the known CD4bs bNAbs. Overall, the M1214_N1 epitope was distinct from the CD4bs by reaching to a long corridor region between glycans at N197 and N386, and contacting V2 at the Env apex side, away from the CD4bs. The contacts of M1214_N1 with the N197 glycan could restrict the movement of the V3 loop from the neighboring protomer (Figure 5F). Together with its contacts on the V1V2 β barrel, the binding of M1214_N1 stabilized the "closed" conformation of the prefusion Env trimer, as evidenced by reduced binding of mAbs that preferentially recognize the "open" Env conformation, such as anti-V3 crown mAbs 447-52D (Gorny et al., 1992) and AD358_b7 (Jia et al., 2016), as well as mAb 17b (Kwong et al., 1998) that recognizes the co-receptor-binding site (CoRbs). As expected, the bNAb VRC01 Fab also stabilized the SOSIP "closed" conformation to a similar degree, whereas the 2-domain soluble CD4 (sCD4) induced the "open" Env conformation that markedly enhanced the binding by 17b but no or minimum enhancement of binding by the anti-V3 crown mAbs

A

M4008 bNAbs

```
Heavy Chain
           ----------FR1----------____CDR1______-----FR2------______CDR2_______--------------FR3---------------_______CDR3______-----FR4-------
VH1-69     QVQLVQSGAEVKKPGSSVKVSCKASGGTFSSYAIS  WVRQAPGQGLEWMGGIIPIFGTANYAQKFQGRVTITADESTSTAYMELSSLRSEDTAVYYCAR
N1 IgG2    ..QLVQSGAEMKTSGSSVRVSCKDSGGFFPYAGFR  WVRQAPGQGFEWMGGVIPADGTKHYAPKFQARMKMTVVESSRTLYMELRSLTSTDTATYFCARLQCAGFSCEMDSGPFDLWGQGTQVTVPSSGAS
N1.2 IgG   QVQLIQSGPEVRQIGSSVKVSCKAAGGVLSYAAFR  WVRQTPGQKLEWLGGIIPGDGTKNYGQKFQGRVKMTADESSRTLYLEMNNLTPDDTGIYFCARLQCGQSDCQLDSAPYDLWGQGTQVTVPSPVPS
N1.3 IgG   QGQLVQSGAEVRQPGSSVKVSCRASGGSLSDAGFR  WVRQAPGHPFEWMGEIIPGDGTRHVGQKFQGRMTMTVE..RATLSLELKDLKPDDTALYFCARLQCGPSDCQLDSAPYDLWGQGTQVTIPSPVPT
N1.3 IgA   QGQLVQSGAEVRQPGSSVKVSCKASGGSLRDAGFR  WVRQAPGQGFEWMGAIIPGDGSKHVGQKFQGRMTMTVD..RATLQMELKNLKPDDTGLYFCARLQCGPSDCQLDSGPYDLWGQGTQVTIPSPVPR
N1.4 IgA   QVRLLQSGPEVKRSGSSVKVSCKLSGGIMSNLGFR  WVRQAPGQGFEYVGGAVPADGKTATGQKFKDRLKISMDERLREVYMELRSLTPADTADYFCATLKCRDESCELDSGPYGAWGQGTRVTVWS

VH4-61     QVQLQESGPGLVKPSETLSLTCTVSGGSVSSGSYYWSWIRQPPGKGLEWIG YIYYSGSTNYNPSLKSRVTISVDTSKNQFSLKLSSVTAADTAVYYCAR
N2 IgG3    QPRLQESGPGRVKPSETLALTCSVSHFAVS.TSY.WAWIRQRPGKGLEWIG YFHHSGTAKYAPSLRKRVSMSMDTSRNTLSLGLTSMTTADTATYYCARVKIIMEETPVMWFDP  WGPGTLVTVSS

Light Chain
           -----------FR1---------______CDR1______------FR2------__CDR2_--------------FR3----------__CDR3_____----FR4-----
VK1-5      DIQMTQSPSTLSASVGDRVTITCRASQSISSWLAWYQQKPGKAPKLLIYDASSLESGVPSRFSGSGSGTEFTLTISSLQPDDFATYYCQQYNSYS
N1_K       DIQMTQSPSTVAAFVGGNVTLSCRTSQGVGNRLAWYQQKPGKPPRLLISRASNRHGGVPARFSGGGSGTIFTLTIKGLQSDDFATFFCQQYYDSRET  FGQGSRVMMEKI
N1_K2      DIQMTQSPSTVAAFVGGNVTLSCRTSQGVGNRLAWYQQKPGKAPRLLISRASTRQGGVPARFSGGGSGTLFTLTIRGLQSDDFATFFCQQYYDSRET  FGQGTRVMMEQR
N1_K3      DIQMTQSPSTLAAFVGGNVTLSCRASQGVADRLAWYQQRPGKAPRLLLFRASSRQRGVPSRFSGGGSGTTFTLTIRGLQSDDFATYFCQQYIDTRET  FGQGTRVIMENK

VL3-21     SYVLTQPPS VSVAPGKTARITCGGNNIGSKSVHWYQQKPGQAPVLVIYYDSDRPSGIPERFSGSNSGNTATLTISRVEAGDEADYYCQVWDSSSDH
N2_L       SYELTQAPS VSVATGQTAKIACGGRNIGSRSVHWYQQRPGRAPVVVIYYDDDRAAGIPERFSGSNSGNTATLTISRVEGGDEAVYYCQVWDASGPQVLFGGGTNLIVL
```

M1214 bNAbs

```
Heavy Chain
           ----------FR1----------_____CDR1_____------FR2-----_______CDR2________--------------FR3---------------________CDR3_______----FR4----
VH3-66     EVQLVESGGGLVQPGGSLRLSCAASGFTVSSNYMS  WVRQAPGKGLEWVSVIYSGGSTY  YADSVKGRFTISRDNSKNTLYLQMNSLRAEDTAVYYCAR
N1 IgG1    D.RLFQSGGGVSRPGGSLRVNCGASGFTVRTHYMY  WLRQSPGKGLEWVAFMNSGGSVS  YVDSVRGRFSVSRDNPANAMVLQMDALKIEDTGTYYCARELREAWYGDLRDYSGLDV  WGRGTIVSISS
N1.2 IgG   E.SVEQSGDASSHPGGSLRVYCWASDFTVRTHYMY  WLRQSPGKGLQWVAFMNSGGTTF  YTDSVKGRFSVSRDNPQNAMVLEMNDLKVDDTGIYYCARELREAWSGDLRDYTGLDV  WGRGTTVTISS
N1.2 IgA   D.SVGQSGDASSRPGGSLRVHCWASDFTVRTHYMY  WLRQSPGKGLQWVAFMNSRGATF  YTDSVKGRFSVSRDDPQNAMVLEMNDLKVDDTGIYYCARELREAWSGDLRDYTGLDV  WGRGTTVTISS

VH4-34     QVQLQQWGAGLLKPSETLSLTCAVYGGSFSGYYWS  WIRQPPGKGLEWIGEINHSGSTN  YNPSLKSRVTISVDTSKNQFSLKLSSVTAADTAVYYCAR
N2 IgG1    .VRLVQWGSGLVKPSETLKVTCAAYDVTFSDVYWA  WVRLSPGRGLEWLGEVGQWGPTQ  TNPSFKGRLTLSVDKSLGHYSLTLTKVTAADTATYFFARIVSQVWGTLDL         WSQGTHVRVSS
N2.2 IgG1  .VRLVQWGTGLLKPSETLSITCAVYDMTFGDKYWS  WVRLSPERGLEWIGEIGQWGETH  YNPSVGSRATLGVDRSLSHFSLRLTSVTPADTAIYFFARVDPSRGGSFDL         WSHGTKVLVSS

VH6-1      QVQLQQSGPGLVKPSQTLSLTCAISGDSVSSNSAAWNWIRQSPSRGLEWLGRTYYRSKWYNDYAVSVKSRITINPDTSKNQFSLQLNSVTPEDTAVYYCAR
N3 IgG1    QVQLQESGPGLVKPSQNVSLTCAVSGESITNPYFAFDWIRQSPSRGLEWLGRTYFRSKWYYEHAKSLKGRLHISVDTSKNQVSLQMDSVTHEDTAIYFCTRGGYENRWWPHDDAFNGLELWGQGTPVIVSS

Light Chain
           ----------FR1----------_________CDR1________------FR2------__CDR2_-----------FR3-------------____CDR3___---FR4----
VL2-11     QSALTQ PRSVSGSPGQSVTISC  TGTSSDVGGYNYVSWYQQHPGKAPKLMIYDVSKRPSGVPDRFSGSKSGNTASLTISGLQAEDEADYYCCSYAGSYTF
N1         QSALAQ PPSVSGSPGQSVTITC  TGINDYGAAYKFVSWYQQHPGKEPRLIMKNVKDRWSVTPNRFSGSTSGNTASLTISNLQSDDEAQYFCAVYAGGFTFPRLGGGTKLSVL
N1.2       QSALTQ PPSVSGSPGQSVTIPC  TGINDRGDGKKFISWYQQHPGKEPRLIMKDVKHRWSTIPDRFSGSRSGNTASLIISGLKSEDEALYFCAVYVGGFVFPRLGGGTKLTVL

VK2-24     DIVMTQTPLSSPVTLGQPASISCRSSQSLVHSDGNTYLSWLQQRPGQPPRLLIYKISNRFSGVPDRFSGSGAGTDFTLKISRVEAEDVGVYYCMQATQFP
N2         AIVLSQAPLSVPVTLGQPASIFCKSDRSLVHSNGNTYLSWLQQRPGQPPRLLISRVSARVSGVPDKFSGSGAGTDFILNISRVDAVDVAVYYCMQGSEFPWT   FGQGSKLDIK
N2.2       QIVLTQNPLSLSGTPGQPASISCSSSQSLVHNNGNTYLSWLHQRPGQPPRVLIVGISSRLSGVPDRFRGGGAVTEFTLNITRVEPVDVGMYYCMQGSQFPWT   FGQGTRLDIK

VL1-44     QSVLTQ PPSASGTPGQRVTISC  SGSSSNIGS NTVNWYQQLPGTAPKLLIYSNNQRPSGVPDRFSGSKSGTSASLAISGLQSEDEADYYCAAWDDSLNG
N3         QSVLTQ PPSLSAAPGHKINISC  SGGGSNIGS RTVHWYQQLPSRAPKLIIDHDHRRPSGVSDRFTASKSGTSASLFISKLQAADEATYYCAAWDDSLSGFVFGGGTRVVVT
```

B

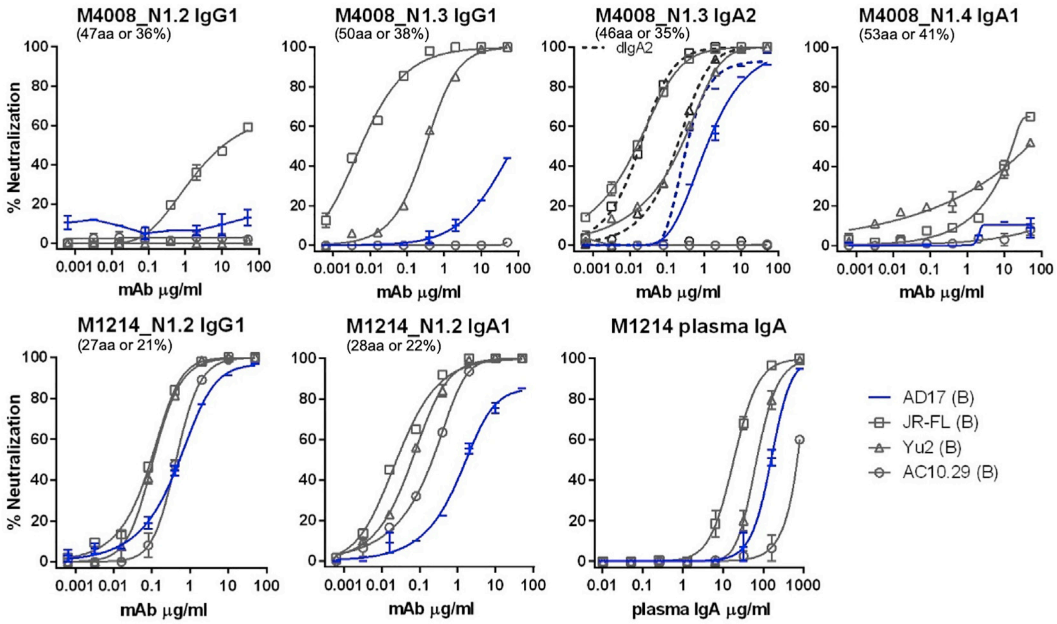

Figure 3. Sequence Alignment and Neutralization Assessment of Representative bNAb Lineage Members

(A) The deduced aa sequences of the variable region of bNAbs and representative lineage members are shown. Framework (FR) and complementarity-determining regions (CDRs) are indicated above the sequence alignment. The top sequence in each group represents the deduced germline V-gene sequence. The dot

(legend continued on next page)

(Figure S3). An analysis of HIV-1 Env sequence from over 5,000 circulating strains indicated that the M1214_N1 contact residues on gp120 are well conserved (mean entropy score of 0.8) (Table S2), comparable to the conservation scores of the epitopes targeted by CD4bs and fusion peptide bNAbs (Chuang et al., 2019). The conservation of this epitope explained the > 60% neutralization breadth for the M1214_N1 clone. However, the V2 contacts (entropy scores of 0.365–0.806) to M1214_N1 that were less conserved than those in the CD4bs including the CD4-binding loop (entropy scores of 0.787–0.992, excluding L369; Table S2) likely accounted for the lower neutralization breadth (65%) of M1214_N1 compared with that of the best CD4bs bNAbs (> 90%) such as the VRC01 class (note that L369 is not a contact residue for VRC01) (Zhou et al., 2010).

DISCUSSION

The VSV_{AD17} probes employed here identified five HIV-1 bNAbs, providing a proof-of-concept that the VSV particles with functional membrane-embedded HIV-1 Env trimers are effective at probing B cells for bNAb isolation. Given the advantageous properties of the VSV_{ENV} particles that are portable, can be conveniently propagated to high titers, and have the flexibility to swiftly insert various HIV-1 Envs including T/F Envs, it becomes feasible to isolate bNAbs from a large number (i.e., dozens) of donor samples. The probe-identified bNAbs were then used as references to identify other bNAb lineage members from the NGS data of donor BCR repertoires. The repertoire analysis identified two bNAb lineages, M4008_N1 and M1214_N1, that were highly unusual in that they class-switched to both IgG and IgA, using both primary and secondary class-switching mechanisms. The HIV-1 IgA bNAbs have not been identified until now, mainly due to paucity of IgA responses to HIV-1, as compared with that of IgG (Mestecky et al., 2009). Therefore, previous bNAb isolation efforts and NGS analyses of BCR repertoires have focused on IgG^+ B cells and missed IgA^+ B cells (Doria-Rose et al., 2014; Wu et al., 2015; Wu et al., 2011). Here, we included the IgA transcripts in BCR repertoire analyses, and our positive findings are also a result of the fact that the two studied donors had mounted significant IgA bNAb responses to HIV-1. In contrast to previous studies where IgG bNAbs were artificially converted to IgA for functional analysis (Duchemin et al., 2018; Tudor et al., 2012; Wolbank et al., 2003), these IgA bNAbs were naturally produced in patients during HIV-1 chronic infection, thus making it feasible to assess naturally produced IgA bNAbs for protection and determine whether IgA bNAbs function similarly to or differently from their IgG counterparts at the mucosal portal of infection. This also provided a physiologically relevant example for class-switching from IgG to IgA, as a general immunological mechanism, in human antibodies with known specificity and function. This finding inspired subsequent and relevant questions such as whether the mechanism of IgG class-switching to IgA is common for HIV-1 bNAbs, and what factors determine which bNAb clones class-switch to both IgG and IgA. More importantly, epitope mapping and a cryo-EM structure revealed that these two bNAb lineages targeted two different epitopes, one at the V3 loop but independent of glycans and the other at the V2V5 corridor. Because previous antibodies targeting the V3 crown of HIV-1 gp120 can only neutralize tier 1 viruses and are weak against tier 2 viruses (Andrabi et al., 2013; Li et al., 2015), the V3 epitope defined by M4008_N1 and its clonal variants that conferred 42% (with $IC_{50} < 50$ μg/mL) or 23% (with $IC_{50} < 1$ μg/mL) of tier 2 virus neutralization is highly relevant for immunogen design targeting the V3 loop (Zolla-Pazner et al., 2015), an immunodominant region of HIV-1 Env. Further structural analysis of M4008_N1 in complex with HIV-1 Env will help determine the specific antibody residues that contact V3 and confer effective tier 2 virus neutralization. Because the V3 crown epitope and the V2V5 corridor epitope reported here were distinct from those previously described, both added a site of vulnerability on the HIV-1 Env and thus were relevant to Env immunogen design. It is still unclear whether these specific sites are intrinsic factors or are coincident with the unusual class-switching to both IgG and IgA. With more such bNAb clones to be discovered and characterized, the intrinsic properties associated with bNAb clones that class-switch to both IgG and IgA will be unveiled.

STAR★METHODS

Detailed methods are provided in the online version of this paper and include the following:

- KEY RESOURCES TABLE
- RESOURCE AVAILABILITY
  - Lead Contact
  - Materials Availability
  - Data and Code Availability
- EXPERIMENTAL MODEL AND SUBJECT DETAILS
  - Cell Lines
  - Patient Samples
- METHOD DETAILS
  - Antibodies, Plasmids, and Proteins
  - VSV_{AD17} Construct and Probe Preparation
  - Fluorescence Activated Cell Sorting, Single B cell RT-PCR, Antibody Expression and Purification
  - Enzyme-linked Immunosorbent Assay (ELISA)
  - HIV-1 Neutralization Assay
  - 5′ RACE and Next-Generation Sequencing (NGS) of B Cell Receptor (BCR) Repertoires

symbol marks deletion; red residues indicate somatic hypermutations, and magenta residues indicate common somatic hypermutations among lineage members, as shown.

(B) The bNAb lineage members were reconstituted as IgG1, IgA1, IgA2, or dIgA2, and then assessed for neutralization against the indicated Env pseudoviruses. The number and percent of aa difference from the probe-identified bNAb reference is indicated in parenthesis for the heavy-chain variable region of each clonal member. The M4008_N1 clonal members shown were paired with N1_K2 κ chain, and the M1214_N1.2 clonal members shown were paired with N1.2 λ chain. Also shown is the neutralization profile of the purified IgA fraction (polyclonal) from donor M1214 plasma. The VSV_{AD17} probe strain AD17 is highlighted in blue. Neutralization was performed in duplicate wells, and the data shown are means with SEM.

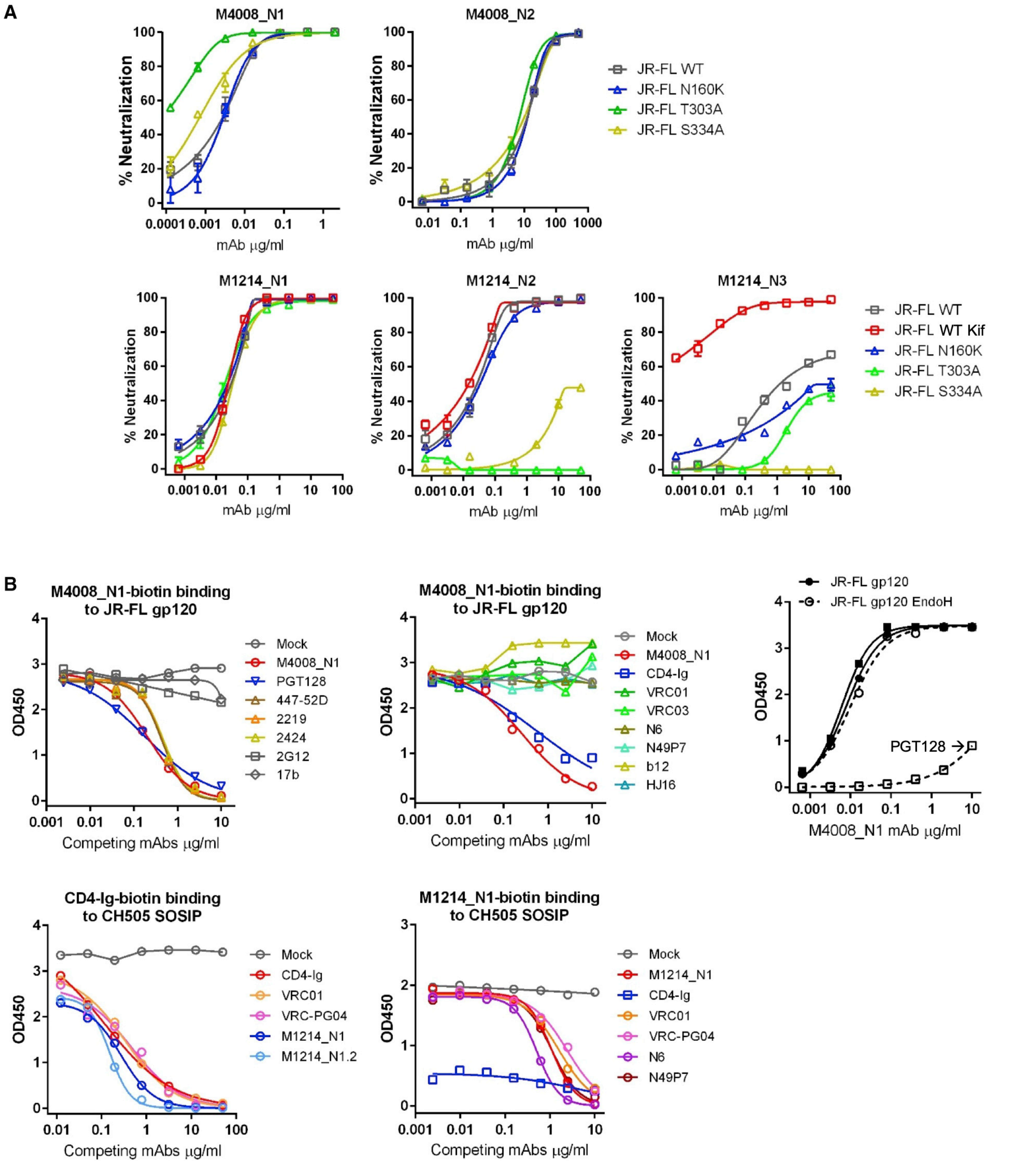

Figure 4. Epitope Mapping of HIV-1 bNAbs

(A) Epitope mapping of bNAbs by comparison of neutralization profiles of each bNAb on the HIV-1 strain JR-FL, as WT, treated with Kifunensine (Kif), or with indicated point mutations. Neutralization was performed in duplicate wells, and the data shown are means with SEM.

(B) Epitope mapping of bNAbs by standard and competition ELISAs. The competition ELISAs were performed with a single concentration of biotinylated reagent (bNAbs or CD4-Ig, as indicated) binding to JR-FL gp120 or CH505 SOSIP. The unlabeled competing mAbs or CD4-Ig were titrated into the ELISAs at increasing concentrations to evaluate the effect on biotinylated reagent binding.

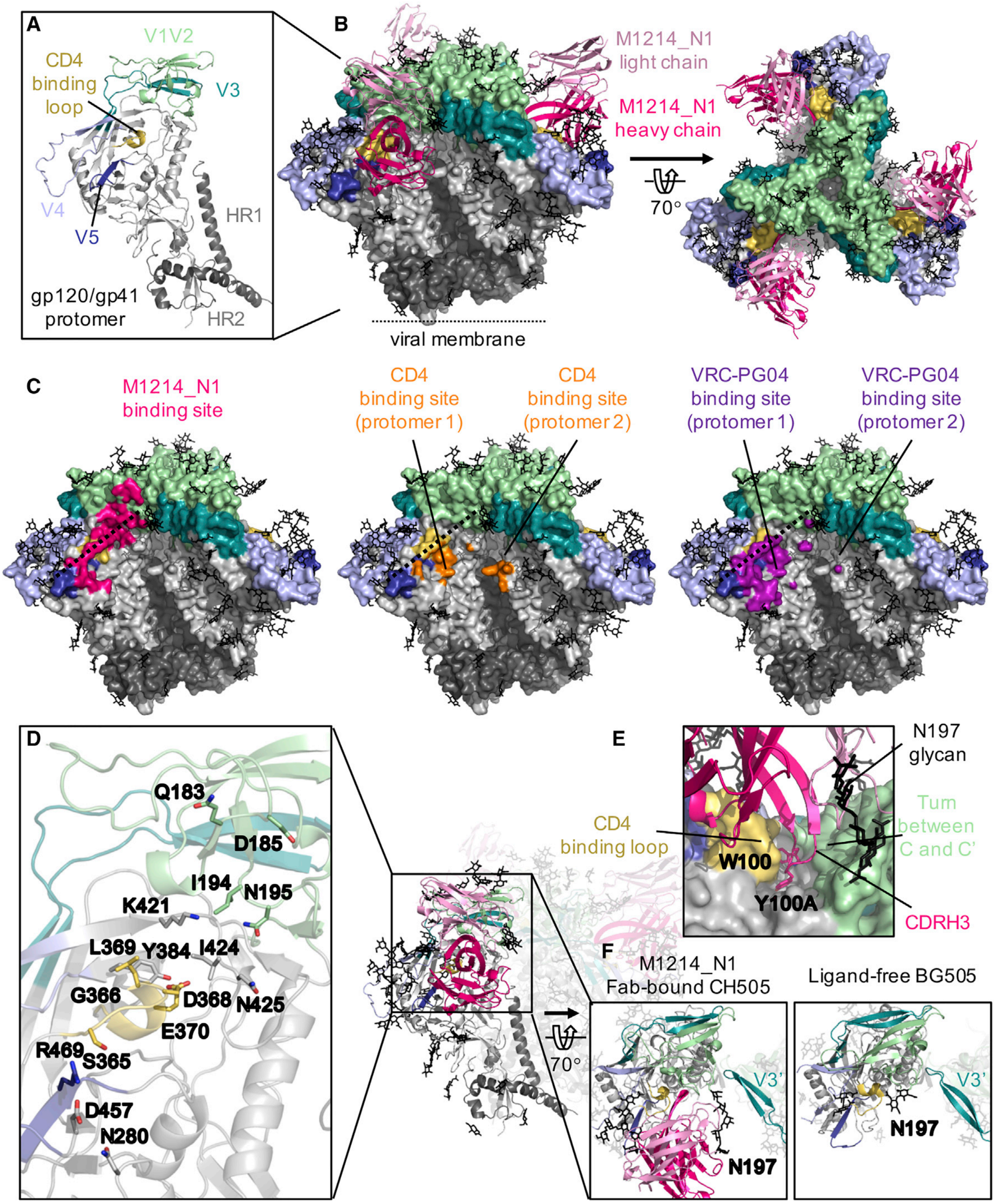

Figure 5. Cryo-EM Structure of M1214_N1 Fab in Complex with CH505 SOSIP Trimer

(A) Structural model of a CH505 gp120/gp41 protomer. The variable (V1-V5) regions and CD4-binding loop in gp120 and HR1 and HR2 helices in gp41 are highlighted, with the same color scheme throughout the figure.

(B) Cryo-EM structure of three M1214_N1 Fabs (ribbon) on a CH505 SOSIP trimer in side (left) and top (right) views. Glycans on the trimer are depicted in black sticks. For clarity, only the Fv domains of M1214_N1 are shown.

(C) Comparison of the binding site of M1214_N1 (hot pink) to that of CD4 (orange) (Protein Data Bank [PDB]: 5U1F) and the CD4bs-directed bNAb VRC-PG04 (purple) (PDB: 3J5M). The dashed line depicts the CD4-binding loop ridge.

(D) Env residues with surface contact areas > 10 $Å^2$ in M1214_N1 binding are shown in sticks.

(E) Close-up view of the pocket accommodating CDR H3 of M1214_N1.

(F) Position change of the N197 glycan on M1214_N1-bound CH505 SOSIP compared with a ligand-free BG505 SOSIP (PDB: 4ZMJ), restricting the movement of the V3 loop from the neighboring protomer (V3′).

- ○ Analysis of NGS Data
- ○ Antibody Lineage Analysis
- ○ M1214_N1 Fab Crystal Structure Determination
- ○ Cryo-EM sample Preparation and Data Acquisition
- ● QUANTIFICATION AND STATISTICAL ANALYSIS

SUPPLEMENTAL INFORMATION

Supplemental Information can be found online at https://doi.org/10.1016/j.chom.2020.03.024.

ACKNOWLEDGMENTS

We thank the participants in the Montreal HIV-1 infection study Cohort and Mario Legault for Cohort coordination. We also thank Dr. Zheng Liu for help with cryo-EM data collections and Dr. Xunqing Jiang for technical assistance. Financial support for this study was provided by the National Institutes of Health (NIH) grants R01 AI122953 to X.W., R37 AI64003 to P.D.B., P01 AI100151 and R01 AI145655 to X.-P.K., R21 AI138024 to Z.S., and R01 AI134824 to K.C.; by the National Center for Advancing Translational Sciences, NIH, grant UL1TR001866 through The Rockefeller University; and by the Bill and Melinda Gates Foundation Collaboration for AIDS Vaccine Discovery (CAVD) grant OPP1146996 to M.S.S. A.F. is recipient of a Canada Research Chair. D.E.K is recipient of a FRQS Merit Award. The funders had no role in study design, data collection and analysis, decision to publish, or preparation of the manuscript.

AUTHOR CONTRIBUTIONS

M.J., R.A.L., P.D.B., and X.W. conceived and designed the study. M.J., R.A.L., Y.G., K.-W.C., R.P., H.L., and E.W. conducted experiments and analyzed data. E.M., K.C., A.F., D.E.K., M.S.S., and D.D.H. contributed critical resources and reagents. L.S., Z.S., and X.-P.K. supervised bioinformatics and structural analyses. L.S., Z.S., X.-P.K., P.D.B., and X.W. wrote the manuscript, on which all authors commented.

DECLARATION OF INTERESTS

The authors declare no competing interests.

Received: December 20, 2019
Revised: February 27, 2020
Accepted: March 26, 2020
Published: April 20, 2020

REFERENCES

Adams, P.D., Afonine, P.V., Bunkóczi, G., Chen, V.B., Davis, I.W., Echols, N., Headd, J.J., Hung, L.W., Kapral, G.J., Grosse-Kunstleve, R.W., et al. (2010). PHENIX: a comprehensive Python-based system for macromolecular structure solution. Acta Crystallogr. D Biol. Crystallogr. *66*, 213–221.

Andrabi, R., Williams, C., Wang, X.H., Li, L., Choudhary, A.K., Wig, N., Biswas, A., Luthra, K., Nadas, A., Seaman, M.S., et al. (2013). Cross-neutralizing activity of human anti-V3 monoclonal antibodies derived from non-B clade HIV-1 infected individuals. Virology *439*, 81–88.

Arnold, K., Bordoli, L., Kopp, J., and Schwede, T. (2006). The SWISS-MODEL workspace: a web-based environment for protein structure homology modelling. Bioinformatics *22*, 195–201.

Baxter, A.E., Niessl, J., Fromentin, R., Richard, J., Porichis, F., Charlebois, R., Massanella, M., Brassard, N., Alsahafi, N., Delgado, G.G., et al. (2016). Single-cell characterization of viral translation-competent reservoirs in HIV-infected individuals. Cell Host Microbe *20*, 368–380.

Burke, V., Williams, C., Sukumaran, M., Kim, S.S., Li, H., Wang, X.H., Gorny, M.K., Zolla-Pazner, S., and Kong, X.P. (2009). Structural basis of the cross-reactivity of genetically related human anti-HIV-1 mAbs: implications for design of V3-based immunogens. Structure *17*, 1538–1546.

Burnett, P.R., VanCott, T.C., Polonis, V.R., Redfield, R.R., and Birx, D.L. (1994). Serum IgA-mediated neutralization of HIV type 1. J. Immunol. *152*, 4642–4648.

Cale, E.M., Gorman, J., Radakovich, N.A., Crooks, E.T., Osawa, K., Tong, T., Li, J., Nagarajan, R., Ozorowski, G., Ambrozak, D.R., et al. (2017). Virus-like particles identify an HIV V1V2 apex-binding neutralizing antibody that lacks a protruding loop. Immunity *46*, 777–791.

Caskey, M., Klein, F., Lorenzi, J.C., Seaman, M.S., West, A.P., Jr., Buckley, N., Kremer, G., Nogueira, L., Braunschweig, M., Scheid, J.F., et al. (2015). Viraemia suppressed in HIV-1-infected humans by broadly neutralizing antibody 3BNC117. Nature *522*, 487–491.

Caskey, M., Klein, F., and Nussenzweig, M.C. (2019). Broadly neutralizing anti-HIV-1 monoclonal antibodies in the clinic. Nat. Med. *25*, 547–553.

Cerutti, A. (2008). The regulation of IgA class switching. Nat. Rev. Immunol. *8*, 421–434.

Chan, K.W., Pan, R., Costa, M., Gorny, M.K., Wang, S., Lu, S., and Kong, X.P. (2018). Structural comparison of human anti-HIV-1 gp120 V3 monoclonal antibodies of the same gene usage induced by vaccination and chronic infection. J. Virol. *92*, e00641–18.

Chaudhuri, J., and Alt, F.W. (2004). Class-switch recombination: interplay of transcription, DNA deamination and DNA repair. Nat. Rev. Immunol. *4*, 541–552.

Chen, V.B., Arendall, W.B., 3rd, Headd, J.J., Keedy, D.A., Immormino, R.M., Kapral, G.J., Murray, L.W., Richardson, J.S., and Richardson, D.C. (2010). MolProbity: all-atom structure validation for macromolecular crystallography. Acta Crystallogr. D Biol. Crystallogr. *66*, 12–21.

Chuang, G.Y., Zhou, J., Acharya, P., Rawi, R., Shen, C.H., Sheng, Z., Zhang, B., Zhou, T., Bailer, R.T., Dandey, V.P., et al. (2019). Structural survey of broadly neutralizing antibodies targeting the HIV-1 Env trimer delineates epitope categories and characteristics of recognition. Structure *27*, 196–206.

Doria-Rose, N.A., Schramm, C.A., Gorman, J., Moore, P.L., Bhiman, J.N., DeKosky, B.J., Ernandes, M.J., Georgiev, I.S., Kim, H.J., Pancera, M., et al.; NISC Comparative Sequencing Program (2014). Developmental pathway for potent V1V2-directed HIV-neutralizing antibodies. Nature *509*, 55–62.

Duchemin, M., Khamassi, M., Xu, L., Tudor, D., and Bomsel, M. (2018). IgA targeting human immunodeficiency virus-1 envelope gp41 triggers antibody-dependent cellular cytotoxicity cross-clade and cooperates with gp41-specific IgG to increase cell lysis. Front. Immunol. *9*, 244.

Edgar, R.C., and Flyvbjerg, H. (2015). Error filtering, pair assembly and error correction for next-generation sequencing reads. Bioinformatics *31*, 3476–3482.

Emsley, P., and Cowtan, K. (2004). Coot: model-building tools for molecular graphics. Acta Crystallogr. D Biol. Crystallogr. *60*, 2126–2132.

Falkowska, E., Le, K.M., Ramos, A., Doores, K.J., Lee, J.H., Blattner, C., Ramirez, A., Derking, R., van Gils, M.J., Liang, C.H., et al. (2014). Broadly neutralizing HIV antibodies define a glycan-dependent epitope on the prefusion conformation of gp41 on cleaved envelope trimers. Immunity *40*, 657–668.

Garlick, R.L., Kirschner, R.J., Eckenrode, F.M., Tarpley, W.G., and Tomich, C.S. (1990). Escherichia coli expression, purification, and biological activity of a truncated soluble CD4. AIDS Res. Hum. Retroviruses *6*, 465–479.

Gong, S., Tomusange, K., Kulkarni, V., Adeniji, O.S., Lakhashe, S.K., Hariraju, D., Strickland, A., Plake, E., Frost, P.A., Ratcliffe, S.J., et al. (2018). Anti-HIV IgM protects against mucosal SHIV transmission. AIDS *32*, F5–F13.

Gorman, J., Soto, C., Yang, M.M., Davenport, T.M., Guttman, M., Bailer, R.T., Chambers, M., Chuang, G.Y., DeKosky, B.J., Doria-Rose, N.A., et al.; NISC Comparative Sequencing Program (2016). Structures of HIV-1 Env V1V2 with broadly neutralizing antibodies reveal commonalities that enable vaccine design. Nat. Struct. Mol. Biol. *23*, 81–90.

Gorny, M.K., Conley, A.J., Karwowska, S., Buchbinder, A., Xu, J.Y., Emini, E.A., Koenig, S., and Zolla-Pazner, S. (1992). Neutralization of diverse human immunodeficiency virus type 1 variants by an anti-V3 human monoclonal antibody. J. Virol. *66*, 7538–7542.

CellPress
OPEN ACCESS

Hall, T.A. (1999). BioEdit: a user-friendly biological sequence alignment editor and analysis program for Windows 95/98/NT. Nucleic Acids Symp. Ser. *41*, 95–98.

Haynes, B.F., Gilbert, P.B., McElrath, M.J., Zolla-Pazner, S., Tomaras, G.D., Alam, S.M., Evans, D.T., Montefiori, D.C., Karnasuta, C., Sutthent, R., et al. (2012). Immune-correlates analysis of an HIV-1 vaccine efficacy trial. N. Engl. J. Med. *366*, 1275–1286.

Horns, F., Vollmers, C., Croote, D., Mackey, S.F., Swan, G.E., Dekker, C.L., Davis, M.M., and Quake, S.R. (2016). Lineage tracing of human B cells reveals the in vivo landscape of human antibody class switching. eLife *5*, e16578.

Huang, J., Ofek, G., Laub, L., Louder, M.K., Doria-Rose, N.A., Longo, N.S., Imamichi, H., Bailer, R.T., Chakrabarti, B., Sharma, S.K., et al. (2012). Broad and potent neutralization of HIV-1 by a gp41-specific human antibody. Nature *491*, 406–412.

Huang, J., Kang, B.H., Pancera, M., Lee, J.H., Tong, T., Feng, Y., Imamichi, H., Georgiev, I.S., Chuang, G.Y., Druz, A., et al. (2014). Broad and potent HIV-1 neutralization by a human antibody that binds the gp41-gp120 interface. Nature *515*, 138–142.

Jia, M., Lu, H., Markowitz, M., Cheng-Mayer, C., and Wu, X. (2016). Development of broadly neutralizing antibodies and their mapping by monomeric gp120 in human immunodeficiency virus type 1-infected humans and simian-human immunodeficiency virus $SHIV_{SF162P3N}$-infected macaques. J. Virol. *90*, 4017–4031.

Kabsch, W. (2010). Xds. Acta Crystallogr. D Biol. Crystallogr. *66*, 125–132.

Kane, M., Yadav, S.S., Bitzegeio, J., Kutluay, S.B., Zang, T., Wilson, S.J., Schoggins, J.W., Rice, C.M., Yamashita, M., Hatziioannou, T., and Bieniasz, P.D. (2013). MX2 is an interferon-induced inhibitor of HIV-1 infection. Nature *502*, 563–566.

Klein, J.S., and Bjorkman, P.J. (2010). Few and far between: how HIV may be evading antibody avidity. PLoS Pathog. *6*, e1000908.

Kleinfelter, L.M., Jangra, R.K., Jae, L.T., Herbert, A.S., Mittler, E., Stiles, K.M., Wirchnianski, A.S., Kielian, M., Brummelkamp, T.R., Dye, J.M., and Chandran, K. (2015). Haploid genetic screen reveals a profound and direct dependence on cholesterol for hantavirus membrane fusion. MBio *6*, e00801.

Kong, R., Xu, K., Zhou, T., Acharya, P., Lemmin, T., Liu, K., Ozorowski, G., Soto, C., Taft, J.D., Bailer, R.T., et al. (2016). Fusion peptide of HIV-1 as a site of vulnerability to neutralizing antibody. Science *352*, 828–833.

Kozlowski, P.A., Black, K.P., Shen, L., and Jackson, S. (1995). High prevalence of serum IgA HIV-1 infection-enhancing antibodies in HIV-infected persons. Masking by IgG. J. Immunol. *154*, 6163–6173.

Kulkarni, V., and Ruprecht, R.M. (2017). Mucosal IgA responses: damaged in established HIV infection - yet, effective weapon against HIV transmission. Front. Immunol. *8*, 1581.

Kwong, P.D., Wyatt, R., Robinson, J., Sweet, R.W., Sodroski, J., and Hendrickson, W.A. (1998). Structure of an HIV gp120 envelope glycoprotein in complex with the CD4 receptor and a neutralizing human antibody. Nature *393*, 648–659.

Lefranc, M.P., Giudicelli, V., Duroux, P., Jabado-Michaloud, J., Folch, G., Aouinti, S., Carillon, E., Duvergey, H., Houles, A., Paysan-Lafosse, T., et al. (2015). IMGT®, the international ImMunoGeneTics information system® 25 years on. Nucleic Acids Res. *43*, D413–D422.

Li, H., Bar, K.J., Wang, S., Decker, J.M., Chen, Y., Sun, C., Salazar-Gonzalez, J.F., Salazar, M.G., Learn, G.H., Morgan, C.J., et al. (2010). High multiplicity infection by HIV-1 in men who have sex with men. PLoS Pathog. *6*, e1000890.

Li, L., Wang, X.H., Williams, C., Volsky, B., Steczko, O., Seaman, M.S., Luthra, K., Nyambi, P., Nadas, A., Giudicelli, V., et al. (2015). A broad range of mutations in HIV-1 neutralizing human monoclonal antibodies specific for V2, V3, and the CD4 binding site. Mol. Immunol. *66*, 364–374.

Liberatore, R.A., Mastrocola, E.J., Cassella, E., Schmidt, F., Willen, J.R., Voronin, D., Zang, T.M., Hatziioannou, T., and Bieniasz, P.D. (2019). Rhabdo-immunodeficiency virus, a murine model of acute HIV-1 infection. eLife *8*, e49875.

Lin, M., Du, L., Brandtzaeg, P., and Pan-Hammarström, Q. (2014). IgA subclass switch recombination in human mucosal and systemic immune compartments. Mucosal Immunol. *7*, 511–520.

Lopez, E., Shattock, R.J., Kent, S.J., and Chung, A.W. (2018). The multifaceted nature of immunoglobulin A and its complex role in HIV. AIDS Res. Hum. Retroviruses *34*, 727–738.

Lu, M., Ma, X., Castillo-Menendez, L.R., Gorman, J., Alsahafi, N., Ermel, U., Terry, D.S., Chambers, M., Peng, D., Zhang, B., et al. (2019). Associating HIV-1 envelope glycoprotein structures with states on the virus observed by smFRET. Nature *568*, 415–419.

Lynch, R.M., Boritz, E., Coates, E.E., DeZure, A., Madden, P., Costner, P., Enama, M.E., Plummer, S., Holman, L., Hendel, C.S., et al.; VRC 601 Study Team (2015). Virologic effects of broadly neutralizing antibody VRC01 administration during chronic HIV-1 infection. Sci. Transl. Med. *7*, 319ra206.

Ma, X., Lu, M., Gorman, J., Terry, D.S., Hong, X., Zhou, Z., Zhao, H., Altman, R.B., Arthos, J., Blanchard, S.C., et al. (2018). HIV-1 Env trimer opens through an asymmetric intermediate in which individual protomers adopt distinct conformations. eLife *7*, e34271.

Macpherson, A.J., McCoy, K.D., Johansen, F.E., and Brandtzaeg, P. (2008). The immune geography of IgA induction and function. Mucosal Immunol. *1*, 11–22.

Magri, G., Comerma, L., Pybus, M., Sintes, J., Llige, D., Segura-Garzon, D., Bascones, S., Yeste, A., Grasset, E.K., Gutzeit, C., et al. (2017). Human secretory IgM emerges from plasma cells clonally related to gut memory B cells and targets highly diverse commensals. Immunity *47*, 118–134.

Maiti, R., Van Domselaar, G.H., Zhang, H., and Wishart, D.S. (2004). SuperPose: a simple server for sophisticated structural superposition. Nucleic Acids Res. *32*, W590–W594.

Mastronarde, D.N. (2005). Automated electron microscope tomography using robust prediction of specimen movements. J. Struct. Biol. *152*, 36–51.

McCoy, A.J., Grosse-Kunstleve, R.W., Adams, P.D., Winn, M.D., Storoni, L.C., and Read, R.J. (2007). Phaser crystallographic software. J. Appl. Cryst. *40*, 658–674.

McLellan, J.S., Pancera, M., Carrico, C., Gorman, J., Julien, J.P., Khayat, R., Louder, R., Pejchal, R., Sastry, M., Dai, K., et al. (2011). Structure of HIV-1 gp120 V1/V2 domain with broadly neutralizing antibody PG9. Nature *480*, 336–343.

Mestecky, J., Moldoveanu, Z., Smith, P.D., Hel, Z., and Alexander, R.C. (2009). Mucosal immunology of the genital and gastrointestinal tracts and HIV-1 infection. J. Reprod. Immunol. *83*, 196–200.

Pan, R., Gorny, M.K., Zolla-Pazner, S., and Kong, X.P. (2015). The V1V2 region of HIV-1 gp120 forms a five-stranded beta barrel. J. Virol. *89*, 8003–8010.

Pegu, A., Borate, B., Huang, Y., Pauthner, M.G., Hessell, A.J., Julg, B., Doria-Rose, N.A., Schmidt, S.D., Carpp, L.N., Cully, M.D., et al. (2019). A meta-analysis of passive immunization studies shows that serum-neutralizing antibody titer associates with protection against SHIV challenge. Cell Host Microbe *26*, 336–346.

Pejchal, R., Doores, K.J., Walker, L.M., Khayat, R., Huang, P.S., Wang, S.K., Stanfield, R.L., Julien, J.P., Ramos, A., Crispin, M., et al. (2011). A potent and broad neutralizing antibody recognizes and penetrates the HIV glycan shield. Science *334*, 1097–1103.

Pettersen, E.F., Goddard, T.D., Huang, C.C., Couch, G.S., Greenblatt, D.M., Meng, E.C., and Ferrin, T.E. (2004). UCSF Chimera–a visualization system for exploratory research and analysis. J. Comput. Chem. *25*, 1605–1612.

Punjani, A., Rubinstein, J.L., Fleet, D.J., and Brubaker, M.A. (2017). cryoSPARC: algorithms for rapid unsupervised cryo-EM structure determination. Nat. Methods *14*, 290–296.

Rohou, A., and Grigorieff, N. (2015). CTFFIND4: Fast and accurate defocus estimation from electron micrographs. J. Struct. Biol. *192*, 216–221.

Rudicell, R.S., Kwon, Y.D., Ko, S.Y., Pegu, A., Louder, M.K., Georgiev, I.S., Wu, X., Zhu, J., Boyington, J.C., Chen, X., et al.; NISC Comparative Sequencing Program (2014). Enhanced potency of a broadly neutralizing HIV-1 antibody in vitro improves protection against lentiviral infection in vivo. J. Virol. *88*, 12669–12682.

Saunders, K.O., Verkoczy, L.K., Jiang, C., Zhang, J., Parks, R., Chen, H., Housman, M., Bouton-Verville, H., Shen, X., Trama, A.M., et al. (2017). Vaccine induction of heterologous tier 2 HIV-1 neutralizing antibodies in animal models. Cell Rep. *21*, 3681–3690.

Scheid, J.F., Mouquet, H., Ueberheide, B., Diskin, R., Klein, F., Oliveira, T.Y., Pietzsch, J., Fenyo, D., Abadir, A., Velinzon, K., et al. (2011). Sequence and structural convergence of broad and potent HIV antibodies that mimic CD4 binding. Science *333*, 1633–1637.

Scheres, S.H. (2012). RELION: implementation of a Bayesian approach to cryo-EM structure determination. J. Struct. Biol. *180*, 519–530.

Schneider, T., Ullrich, R., and Zeitz, M. (1996). The immunologic aspects of human immunodeficiency virus infection in the gastrointestinal tract. Semin. Gastrointest. Dis. *7*, 19–29.

Schoofs, T., Barnes, C.O., Suh-Toma, N., Golijanin, J., Schommers, P., Gruell, H., West, A.P., Jr., Bach, F., Lee, Y.E., Nogueira, L., et al. (2019). Broad and potent neutralizing antibodies recognize the silent face of the HIV envelope. Immunity *50*, 1513–1529.

Schramm, C.A., Sheng, Z., Zhang, Z., Mascola, J.R., Kwong, P.D., and Shapiro, L. (2016). SONAR: a high-throughput pipeline for inferring antibody ontogenies from longitudinal sequencing of B cell transcripts. Front. Immunol. *7*, 372.

Seaman, M.S., Leblanc, D.F., Grandpre, L.E., Bartman, M.T., Montefiori, D.C., Letvin, N.L., and Mascola, J.R. (2007). Standardized assessment of NAb responses elicited in rhesus monkeys immunized with single- or multi-clade HIV-1 envelope immunogens. Virology *367*, 175–186.

Seaman, M.S., Janes, H., Hawkins, N., Grandpre, L.E., Devoy, C., Giri, A., Coffey, R.T., Harris, L., Wood, B., Daniels, M.G., et al. (2010). Tiered categorization of a diverse panel of HIV-1 Env pseudoviruses for assessment of neutralizing antibodies. J. Virol. *84*, 1439–1452.

Sheng, Z., Schramm, C.A., Kong, R., Mullikin, J.C., Mascola, J.R., Kwong, P.D., and Shapiro, L.; NISC Comparative Sequencing Program (2017). Gene-specific substitution profiles describe the types and frequencies of amino acid changes during antibody somatic hypermutation. Front. Immunol. *8*, 537.

Sievers, F., Wilm, A., Dineen, D., Gibson, T.J., Karplus, K., Li, W., Lopez, R., McWilliam, H., Remmert, M., Söding, J., et al. (2011). Fast, scalable generation of high-quality protein multiple sequence alignments using Clustal Omega. Mol. Syst. Biol. *7*, 539.

Spence, J.S., Krause, T.B., Mittler, E., Jangra, R.K., and Chandran, K. (2016). Direct visualization of ebola virus fusion triggering in the endocytic pathway. MBio *7*, e01857–e15.

Tamura, K., Stecher, G., Peterson, D., Filipski, A., and Kumar, S. (2013). MEGA6: molecular evolutionary genetics analysis version 6.0. Mol. Biol. Evol. *30*, 2725–2729.

Thomas, D., Newcomb, W.W., Brown, J.C., Wall, J.S., Hainfeld, J.F., Trus, B.L., and Steven, A.C. (1985). Mass and molecular composition of vesicular stomatitis virus: a scanning transmission electron microscopy analysis. J. Virol. *54*, 598–607.

Tiller, T., Meffre, E., Yurasov, S., Tsuiji, M., Nussenzweig, M.C., and Wardemann, H. (2008). Efficient generation of monoclonal antibodies from single human B cells by single cell RT-PCR and expression vector cloning. J. Immunol. Methods *329*, 112–124.

Tudor, D., Yu, H., Maupetit, J., Drillet, A.S., Bouceba, T., Schwartz-Cornil, I., Lopalco, L., Tuffery, P., and Bomsel, M. (2012). Isotype modulates epitope specificity, affinity, and antiviral activities of anti-HIV-1 human broadly neutralizing 2F5 antibody. Proc. Natl. Acad. Sci. USA *109*, 12680–12685.

Veillette, M., Coutu, M., Richard, J., Batraville, L.A., Dagher, O., Bernard, N., Tremblay, C., Kaufmann, D.E., Roger, M., and Finzi, A. (2015). The HIV-1 gp120 CD4-bound conformation is preferentially targeted by antibody-dependent cellular cytotoxicity-mediating antibodies in sera from HIV-1-infected individuals. J. Virol. *89*, 545–551.

Walker, L.M., Phogat, S.K., Chan-Hui, P.Y., Wagner, D., Phung, P., Goss, J.L., Wrin, T., Simek, M.D., Fling, S., Mitcham, J.L., et al.; Protocol G Principal Investigators (2009). Broad and potent neutralizing antibodies from an African donor reveal a new HIV-1 vaccine target. Science *326*, 285–289.

Walker, L.M., Huber, M., Doores, K.J., Falkowska, E., Pejchal, R., Julien, J.P., Wang, S.K., Ramos, A., Chan-Hui, P.Y., Moyle, M., et al.; Protocol G Principal Investigators (2011). Broad neutralization coverage of HIV by multiple highly potent antibodies. Nature *477*, 466–470.

Waltari, E., Jia, M., Jiang, C.S., Lu, H., Huang, J., Fernandez, C., Finzi, A., Kaufmann, D.E., Markowitz, M., Tsuji, M., and Wu, X. (2018). 5′ rapid amplification of cDNA ends and Illumina MiSeq reveals B cell receptor features in healthy adults, adults with chronic HIV-1 infection, cord blood, and humanized mice. Front. Immunol. *9*, 628.

Wang, H., Barnes, C.O., Yang, Z., Nussenzweig, M.C., and Bjorkman, P.J. (2018). Partially open HIV-1 envelope structures exhibit conformational changes relevant for coreceptor binding and fusion. Cell Host Microbe *24*, 579–592.

Williams, L.D., Ofek, G., Schatzle, S., McDaniel, J.R., Lu, X., Nicely, N.I., Wu, L., Lougheed, C.S., Bradley, T., Louder, M.K., et al. (2017). Potent and broad HIV-neutralizing antibodies in memory B cells and plasma. Sci. Immunol. *2*, eaal2200.

Wolbank, S., Kunert, R., Stiegler, G., and Katinger, H. (2003). Characterization of human class-switched polymeric (immunoglobulin M [IgM] and IgA) anti-human immunodeficiency virus type 1 antibodies 2F5 and 2G12. J. Virol. *77*, 4095–4103.

Wu, X., Hall, S., and Jackson, S. (2003). Tropism-restricted neutralization by secretory IgA from parotid saliva of HIV type 1-infected individuals. AIDS Res. Hum. Retroviruses *19*, 275–281.

Wu, X., Yang, Z.Y., Li, Y., Hogerkorp, C.M., Schief, W.R., Seaman, M.S., Zhou, T., Schmidt, S.D., Wu, L., Xu, L., et al. (2010). Rational design of envelope identifies broadly neutralizing human monoclonal antibodies to HIV-1. Science *329*, 856–861.

Wu, X., Zhou, T., Zhu, J., Zhang, B., Georgiev, I., Wang, C., Chen, X., Longo, N.S., Louder, M., McKee, K., et al.; NISC Comparative Sequencing Program (2011). Focused evolution of HIV-1 neutralizing antibodies revealed by structures and deep sequencing. Science *333*, 1593–1602.

Wu, X., Zhang, Z., Schramm, C.A., Joyce, M.G., Kwon, Y.D., Zhou, T., Sheng, Z., Zhang, B., O'Dell, S., McKee, K., et al.; NISC Comparative Sequencing Program (2015). Maturation and diversity of the VRC01-antibody lineage over 15 years of chronic HIV-1 infection. Cell *161*, 470–485.

Wyatt, R., Kwong, P.D., Desjardins, E., Sweet, R.W., Robinson, J., Hendrickson, W.A., and Sodroski, J.G. (1998). The antigenic structure of the HIV gp120 envelope glycoprotein. Nature *393*, 705–711.

Zan, H., Cerutti, A., Dramitinos, P., Schaffer, A., and Casali, P. (1998). CD40 engagement triggers switching to IgA1 and IgA2 in human B cells through induction of endogenous TGF-beta: evidence for TGF-beta but not IL-10-dependent direct S mu–>S alpha and sequential S mu–>S gamma, S gamma–>S alpha DNA recombination. J. Immunol. *161*, 5217–5225.

Zheng, S.Q., Palovcak, E., Armache, J.P., Verba, K.A., Cheng, Y., and Agard, D.A. (2017). MotionCor2: anisotropic correction of beam-induced motion for improved cryo-electron microscopy. Nat. Methods *14*, 331–332.

Zhou, M., and Ruprecht, R.M. (2014). Are anti-HIV IgAs good guys or bad guys? Retrovirology *11*, 109.

Zhou, T., Georgiev, I., Wu, X., Yang, Z.Y., Dai, K., Finzi, A., Kwon, Y.D., Scheid, J.F., Shi, W., Xu, L., et al. (2010). Structural basis for broad and potent neutralization of HIV-1 by antibody VRC01. Science *329*, 811–817.

Zhou, T., Doria-Rose, N.A., Cheng, C., Stewart-Jones, G.B.E., Chuang, G.Y., Chambers, M., Druz, A., Geng, H., McKee, K., Kwon, Y.D., et al. (2017). Quantification of the impact of the HIV-1-glycan shield on antibody elicitation. Cell Rep. *19*, 719–732.

Zhou, T., Zheng, A., Baxa, U., Chuang, G.Y., Georgiev, I.S., Kong, R., O'Dell, S., Shahzad-Ul-Hussan, S., Shen, C.H., Tsybovsky, Y., et al. (2018). A neutralizing antibody recognizing primarily N-linked glycan targets the silent face of the HIV envelope. Immunity *48*, 500–513.

Zhu, P., Chertova, E., Bess, J., Jr., Lifson, J.D., Arthur, L.O., Liu, J., Taylor, K.A., and Roux, K.H. (2003). Electron tomography analysis of envelope

CellPress
OPEN ACCESS

glycoprotein trimers on HIV and simian immunodeficiency virus virions. Proc. Natl. Acad. Sci. USA *100*, 15812–15817.

Zhu, P., Liu, J., Bess, J., Jr., Chertova, E., Lifson, J.D., Grisé, H., Ofek, G.A., Taylor, K.A., and Roux, K.H. (2006). Distribution and three-dimensional structure of AIDS virus envelope spikes. Nature *441*, 847–852.

Zolla-Pazner, S., Cohen, S.S., Boyd, D., Kong, X.P., Seaman, M., Nussenzweig, M., Klein, F., Overbaugh, J., and Totrov, M. (2015). Structure/function studies involving the V3 region of the HIV-1 envelope delineate multiple factors that affect neutralization sensitivity. J. Virol. *90*, 636–649.

STAR★METHODS

KEY RESOURCES TABLE

REAGENT or RESOURCE	IDENTIFIER	SOURCE
Antibodies		
b12, HJ16, VRC01, VRC03, VRC-PG04, N6, 17b, PGT128, 2G12, 2-domain sCD4	NIH AIDS Reagent Program	N/A
N49P7	Institute of Human Virology, University of Maryland	N/A
447-52D, 2219, 2424	New York Univeristy	N/A
AD358_b7	ADARC	N/A
HRP-conjugated goat anti-human IgG Fc	Jackson ImmunoResearch	Cat#109-035-098; RRID: AB_2337586
Chemicals, Peptides, Recombinant Proteins and Biosensors		
JR-FL and Yu2 gp120	ADARC	N/A
Yu2 gp140 foldon	ADARC	N/A
CH505 SOSIP gp140	Duke Human Vaccine Institute	N/A
3,3′,5,5′-tetramethylbenzidine (TMB)	ThermoFisher Scientific	Cat#22311
ExpiFectamine 293 Transfection Kit	ThermoFisher Scientific	Cat#A14524
Fugene 6	Promega	Cat#E2691
Pierce Protein A Agarose	ThermoFisher Scientific	Cat#20334
CaptureSelect IgA Affinity Matrix	Life Technology	Cat#194288010
Peptide M agarose	InvivoGen	Cat#gel-pdm-5
Endoglycosidase H	New England Biolabs	Cat#P0702L
Virus Strains		
120 HIV-1 Env virus panel	Harvard Medical School	N/A
Biological Samples		
plasma and PBMC of donor M4008	Montreal HIV infection cohort	M4008
plasma and PBMC of donor M1214	Montreal HIV infection cohort	M1214
Critical Commercial Assays		
Site-directed mutagenesis	GM Biosciences	N/A
Luciferase Assay System	Promega	Cat#E1501
Oligotex Direct mRNA mini kit	QIAGEN	Cat#72022
SMARTer Pico PCR cDNA Synthesis Kit	Clontech	Cat#634928
Deposited Data		
M4008_N1, M4008_N1.2, M4008_N1.3, M4008_N1.4, M4008_N2	This paper	GenBank accession # MT110156-MT110165
M1214_N1, M1214_N1.2, M1214_N2, M1214_N2.2, M1214_N3	This paper	GenBank accession # MT110166-MT110176
Crystal structure of M1214_N1 Fab	This paper	PDB ID: 6VU2
Cryo-EM structure of M1214_N1 Fab in complex with CH505 SOSIP	This paper	EMD-21456; PDB ID: 6VY2
Illumina MiSeq sequencing of donor M4008 B cell receptor transcripts	This paper	Donor M4008 MiSeq reads have been deposited to NCBI SRA database under accession SRP250531.
Illumina MiSeq sequencing of donor M1214 B cell receptor transcripts	Waltari et al., 2018	Donor M1214 MiSeq reads have been deposited to NCBI SRA databases under accession SRP111345.
Experimental Models: Cell Lines		
Human: 293T cells	ATCC	Cat#CRL-11268; RRID: CVCL_1926
Human: 293T-furin cells	This paper	N/A
Human: Expi293F cells	Thermo Fisher	Cat#A14527; RRID: CVCL_D615

(*Continued on next page*)

CellPress
OPEN ACCESS

Continued

REAGENT or RESOURCE	IDENTIFIER	SOURCE
Human: TZM-bl cells	NIH AIDS Reagent Program	Cat#8129; RRID: CVCL_B478
Recombinant DNA		
Human IgA1, IgA2 heavy chain construct	This paper	N/A
Human J chain construct	This paper	N/A
Software and Algorithms		
XDS software package	Kabsch, 2010	http://xds.mpimf-heidelberg.mpg.de/
Coot	Emsley and Cowtan, 2004	https://www2.mrc-lmb.cam.ac.uk/personal/pemsley/coot/
Pymol	Schrödinger	https://pymol.org
Phenix	Adams et al., 2010	https://www.phenix-online.org/
PRISM 7	GraphPad Software	https://www.graphpad.com/scientific-software/prism/
Dendroscope 3	Daniel H. Huson	http://dendroscope.org
BioEdit v7.2.5	Hall, 1999	https://bioedit.software.informer.com/
IMGT	Lefranc et al., 2015	http://www.imgt.org

RESOURCE AVAILABILITY

Lead Contact

Further information and requests for resources and reagents should be directed to and will be fulfilled by the Lead Contact, Xueling Wu (xw2702@cumc.columbia.edu).

Materials Availability

Expression plasmids encoding VSV_{AD17}-mNG and bNAbs, including the representative bNAb lineage members, will be deposited to the NIH AIDS Reagent Program. The 293T-furin cell line with elevated human furin expression will be deposited to the NIH AIDS Reagent Program as well.

Data and Code Availability

Sequences of M4008 and M1214 bNAbs and their NGS-derived heavy and light chains of variable region are available in GenBank under accession # MT110156-MT110176. The NGS data used in this study have been deposited in the NCBI Sequence Read Archive (https://www.ncbi.nlm.nih.gov/sra) under accession SRP250531 for M4008 and SRP111345 for M1214. The crystal structure of M1214_N1 Fab has been deposited in the Protein Data Bank (PDB: 6VU2). The Cryo-EM reconstruction of M1214_N1 Fab in complex with CH505 SOSIP has been deposited in the Electron Microscopy Data Bank as EMD-21456 and the Protein Data Bank (PDB: 6VY2).

EXPERIMENTAL MODEL AND SUBJECT DETAILS

Cell Lines

Human embryonic kidney 293 cell line, of which the sex is female, is the parental cell for 293T and Expi293F cell lines. 293T was obtained from ATCC and maintained as adherent cells in complete DMEM medium at 37°C. 293T is highly transfectable and contains SV40 T-antigen. The full-length human furin gene was synthesized and cloned into a CSIB-based vector (Kane et al., 2013), which was then used to transduce 293T cells; single cell clones were selected in 5 μg/mL blasticidin, and those with elevated human furin expression (293T-furin) were expanded and maintained as adherent cells in complete DMEM medium with 5 μg/mL blasticidin at 37°C. Expi293F was obtained from Thermo Fisher and adapted to suspension culture in Expi293 Expression Medium at 37°C. TZM-bl, of which the sex is female, was obtained from the NIH AIDS Reagent Program and maintained as adherent cells in complete DMEM medium at 37°C. TZM-bl is a HeLa cell line that expresses CD4 receptor and CXCR4 and CCR5 chemokine co-receptors; the cell line also expresses luciferase and β-galactosidase under the control of the HIV-1 promoter, hence is useful to assay in-vitro HIV-1 infection.

Patient Samples

The two HIV-1^+ plasma and PBMC samples were obtained from the Montreal HIV infection cohort (Baxter et al., 2016; Veillette et al., 2015). The infection time for both donors was over 20 years, and the donors were not on antiretroviral therapy at the time of sampling. We worked with de-identified specimens that contained no information on the age or gender of the patients. All participant samples were collected under Institutional Review Board (IRB) approved protocols and with written informed consent obtained prior to enrollment.

METHOD DETAILS

Antibodies, Plasmids, and Proteins

The anti-CD4bs bNAbs, b12, HJ16, VRC01, VRC03, VRC-PG04 and N6, the anti-co-receptor-binding site (CoRbs) mAb 17b, the anti-V3 stem glycan bNAb PGT128, and the anti-gp120 glycan bNAb 2G12 were obtained through the NIH AIDS Reagent Program, as contributed by Drs. Dennis Burton, Carlos Barbas, Antonio Lanzavecchia, John Mascola, Mark Connors and James Robinson, by International AIDS Vaccine Initiative (IAVI), and by Division of AIDS, NIAID, NIH. The anti-CD4bs bNAb N49P7 was kindly provided by Dr. Mohammad Sajadi. The anti-V3 crown mAbs 447-52D, 2219, and 2424 were provided by Drs. Susan Zolla-Pazner and Miroslaw Gorny, and the anti-V3 crown mAb AD358_b7 has been described (Jia et al., 2016). The 2-domain sCD4 (sCD4-183) was obtained through the NIH AIDS Reagent Program, from Pharmacia, Inc. (Garlick et al., 1990). The expression plasmids encoding the constant region of human IgG1 heavy chain, kappa chain, and lambda chain have been described (Tiller et al., 2008). The constant region sequences of human IgA1 and IgA2 heavy chains as well as the human J chain gene were synthesized and cloned into pcDNA3.1 (Invitrogen). Likewise, the membrane bound human IgG1 heavy chain sequences encoding VRC07 and PGT145 were synthesized and cloned into pcDNA3.1. The CD4-Ig expression plasmid was provided by Dr. Joseph Sodroski, and the CD4-Ig fusion protein was expressed by transient transfection of 293F cells and purified with Pierce recombinant protein A agarose (Thermo Fisher Scientific). The JR-FL gp120 was described (Jia et al., 2016). The expression plasmids encoding Yu2 gp120 and Yu2 gp140 foldon were provided by Drs. Joseph Sodroski and Richard Wyatt, respectively, and the proteins were expressed by transient transfection of 293F cells and purified via His-tag with HisTalon gravity column (Clontech, Mountain View, CA). The CH505 SOSIP trimer was purchased from the Protein Production Facility at Duke Human Vaccine Institute.

VSV_{AD17} Construct and Probe Preparation

As described (Liberatore et al., 2019), the plasmids encoding full-length VSV genome (pVSV-FL) as well as individual VSV genes N, P, L, and G were purchased from Kerafast (Boston, MA). The plasmid encoding the HIV-1 strain AD17 was obtained from the NIH AIDS Reagent Program, as contributed by Drs. Beatrice Hahn and George Shaw. The chimeric Env was generated using overlap-extension PCR, in which the extracellular and transmembrane domains of AD17 Env were fused to the cytoplasmic tail of VSV-G. The chimeric Env DNA fragment was inserted into pVSV-FL precisely in place of the VSV-G to generate $pVSV_{AD17}$. A plasmid bearing VSV cDNA encoding a monomeric NeonGreen-phosphoprotein P fusion protein (mNG-P) has been described (Kleinfelter et al., 2015; Spence et al., 2016). Here, the VSV-G in pVSV-mNG-P was replaced with the chimeric Env DNA fragment to generate $pVSV_{AD17}$-mNG-P. The VSV_{AD17} virus was rescued by infecting 293T cells with T7-expressing vaccinia (vTF7-3) at a MOI of 5, followed by transfection with $pVSV_{AD17}$ (or $pVSV_{AD17}$-mNG-P) and plasmids encoding VSV-N, P, L, and G under the control of a T7 promotor. Supernatant was harvested in 48 h, slowly filtered (0.22 μm) to remove vaccinia and plaque purified on GHOST.R5 cells. Plaque purified VSV_{AD17} was expanded in GHOST.R5 cells, filtered (0.22 μm), and stored in aliquots at −80°C. To prepare VSV_{AD17}-PE, an aliquot of VSV_{AD17} was used to infect a T-75 flask of GHOST.R5 cells; at 24 h after infection, 20 mL of freshly collected VSV_{AD17} supernatant was filtered (0.22 μm) and pelleted by ultracentrifugation; the viral pellet was then labeled with PE (Abcam, Cambridge, MA). To prepare VSV_{AD17}-mNG, an aliquot of VSV_{AD17}-mNG was used to infect a T-75 flask of pVSV-G transfected GHOST.R5 cells; at 24 h after infection, the VSV_{AD17}-mNG (decorated with VSV-G) supernatant was filtered (0.22 μm) and used to infect (single-round infection) a T-75 flask of pre-seeded 293T-furin cells; at 24 h after infection, the 293T-furin cells were inspected under green fluorescent microscope for > 90% confluence and > 90% green fluorescence; 20 mL of freshly collected VSV_{AD17}-mNG supernatant was filtered (0.22 μm) and directly used to stain B cells.

Fluorescence Activated Cell Sorting, Single B cell RT-PCR, Antibody Expression and Purification

Patient or blood donor PBMC samples were pre-sorted for B cells by FACS. Briefly, donor PBMCs were stained with anti-human CD3-PE-CF594 (BD Biosciences, San Jose, CA), CD19-PE-Cy7 (BioLegend, San Diego, CA), and CD20-APC-Cy7 (BioLegend), and the CD3-CD19+ B cells were bulk sorted. In addition, live/dead yellow stain (Invitrogen) was used to exclude dead cells. The bulk sorted B cells were then re-stained with anti-human CD3-PE-CF594, CD19-PE-Cy7, CD20-APC-Cy7, with additional staining of IgM-V450 (BD Biosciences), IgG-FITC (BD Biosciences) and VSV_{AD17}-PE, or IgM-V450 and CD27-PerCP-Cy5.5 (BD Biosciences) with VSV_{AD17}-mNG. Fluorescence compensation was performed with anti-mouse Ig κ beads (BD Biosciences) stained with each antibody in a separate tube. After washing, cells were analyzed and sorted using a multi-laser MoFlo sorter (Beckman Coulter, Jersey City, NJ) contained with Biosafety Level 3 standards; single cells were sorted into 96-well PCR plates containing 20 μL lysis buffer, consisting of 5 μL 5 × first-strand buffer (Invitrogen), 1.25 μL 0.1 M DTT, 0.5 μL RNaseOut, and 0.0625 μL Igepal (Sigma, St. Louis, MO). The total content of cells passing through the sorter was analyzed with FlowJo (TreeStar, Cupertino, CA). The PCR plates with sorted cells were frozen on dry ice and stored at −80°C.

From each sorted cell, the variable regions of antibody heavy and light chains were amplified by RT-PCR as described (Jia et al., 2016; Tiller et al., 2008). Briefly, frozen plates with single B cell RNA were thawed at room temperature, and RT was carried out by adding into each well 3 μL random hexamers at 150 ng/μL (Gene Link, Hawthorne, NY), 2 μL dNTP (each at 10 mM), and 1 μL SuperScript III (Invitrogen), followed by 42°C for 10 min, 25°C for 10 min, 50°C for 60 min and 94°C for 5 min. After RT, 25 μL water was added to each well to dilute cDNA. The heavy and light chain variable regions were amplified independently by nested PCR in 50 μL, using 5 μL cDNA as template, HotStarTaq Plus DNA polymerase (QIAGEN) and primers or primer mixes as described (Scheid et al., 2011; Tiller et al., 2008). Cycler parameters were 94°C for 5 min, 50 cycles of 94°C for 30 s, 52-55°C for 30 s, and 72°C for 1 min, followed by 72°C for 10 min. The PCR amplicons were sequenced and analyzed using IMGT/V-QUEST (http://www.imgt.org/).

Selected PCR sequences that gave productive heavy or light chain rearrangements were reamplified with custom primers containing unique restriction digest sites and cloned into the corresponding human IgG1, IgA1, or IgA2 heavy and light chain expression vectors. Full-length human IgG1, IgA1, or IgA2 proteins were expressed by co-transfecting 293F cells (Invitrogen) with equal amounts of paired heavy and light chain plasmids (along with the human J chain plasmid for dimeric IgA) and purified using Pierce recombinant protein A agarose (Thermo Fisher Scientific) for IgG1 or CaptureSelect IgA Affinity Matrix (Life Technology) for IgA. For each batch of purified dimeric IgA, an analytic size-exclusion chromatography (SEC) was performed to assure > 90% purity. To purify IgA from donor plasma, 1 mL of donor plasma was first passed through a Pierce Protein G UltraLink Column (Thermo Fisher Scientific) to bind IgG, and the IgA in the flow through was then purified using peptide M agarose (InvivoGen).

Enzyme-linked Immunosorbent Assay (ELISA)

As previously described (Jia et al., 2016; Wu et al., 2010), ELISA plates were coated with HIV-1 gp120 at 2 μg/mL or gp140 at 5 μg/mL in PBS at 4°C overnight. For liganded gp140, sCD4, VRC01 Fab, or M1214_N1 Fab was incubated with the same amount of unliganded gp140 in 50 μL of PBS at 37°C for 30 min before used to coat plates at a final concentration of 1 μg/mL in PBS. After blocking with 1% BSA in PBS at 37°C for 1 h, serially diluted antibodies were incubated at 37°C for 1 h. Horseradish peroxidase (HRP)-conjugated goat anti-human IgG Fc antibody (Jackson ImmunoResearch Laboratories Inc., West Grove, PA) was added at 37°C for 1 h. All volumes were 100 μL/well except 200 μL/well for blocking. Plates were washed between each step with 0.1% Tween 20 in PBS, developed with 3,3′,5,5′-tetramethylbenzidine (TMB) (Kirkegaard & Perry Laboratories) and read at 450 nm. For competitive ELISA, serially diluted competitor antibodies or CD4-Ig were added in 50 μL of blocking buffer, followed by adding 50 μL of biotin-labeled antibodies or CD4-Ig at one pre-determined concentration. After incubation at 37°C for 1 h, 250 ng/mL of streptavidin-HRP (Sigma) was added at room temperature for 30 min. Plates were then developed with TMB and read at 450 nm.

HIV-1 Neutralization Assay

Antibody neutralization was assessed based on the single-round infection assay of TZM-bl cells with HIV-1 Env pseudoviruses as described (Jia et al., 2016; Seaman et al., 2010; Seaman et al., 2007). Mutant Envs were generated by TagMaster Site-Directed Mutagenesis Kit (GM Biosciences, Frederick, MD) and tested along with the corresponding wild-type virus. Briefly, 50 or 100 μL of antibody-virus mixture was incubated at 37°C for 30 min in duplicate wells before the addition of TZM-bl cells. To keep assay conditions constant, sham medium was used in place of antibody or plasma in control wells. Infection levels were determined in 2 days with Bright-Glo luciferase assay system (Promega, Madison, WI). Neutralization curves were fitted to a 5-parameter nonlinear regression analysis through Prism 6.0 (GraphPad Software, La Jolla, CA) and the data were plotted as means with SEM (standard error of mean). The antibody concentration or plasma reciprocal dilution required to inhibit infection by 50% was reported as IC_{50} or ID_{50}. Neutralization sensitivity was also color-coded for each tested Env in a dendrogram depicting the gp160 protein sequence distance. Briefly, the HIV-1 gp160 protein sequences of 120 isolates used in the neutralization assay were aligned using ClustalW in BioEdit (https://bioedit.software.informer.com/). The aligned protein sequences were submitted to Protdist and Neighbor phylogenetic tree in BioEdit; the tree was displayed with Dendroscope (http://dendroscope.org) and then color-coded according to the neutralization sensitivity.

5′ RACE and Next-Generation Sequencing (NGS) of B Cell Receptor (BCR) Repertoires

As described (Waltari et al., 2018), cellular mRNA was extracted from the donor PBMCs using the Oligotex Direct mRNA Mini Kit (QIAGEN). For 5′ RACE cDNA synthesis, each 10 μL mRNA was mixed with 1 μL Oligo $dT_{12\text{-}18}$ at 12 μM (Life Technologies) at 70°C for 1 min and then −20°C for 1 min, followed by addition of 1 μL SMARTer Oligo at 12 μM (Clontech), 4 μL 5x first-strand buffer, 1 μL DTT at 20 mM, 1 μL dNTP at 10 mM each, 1 μL RNaseOUT, and 1-3 μL SuperScript II (Life Technologies). The mixtures were incubated at 42°C for 2 h and then passed through a PCR cleanup spin column (Machery-Nagel). The KAPA HiFi qPCR kit (KAPA Biosystems) with a universal 5′ IIA primer (Clontech) was used to amplify the variable region of μ, γ, α, κ, and λ chains from a single cDNA sample, in combination with a mixture of 3′ primers specific for μ, γ, α, κ, and λ chains. Primers each contained a unique 8 bp Illumina barcode for demultiplexing after Miseq sequencing. The PCR cycling conditions were 98°C for 45 s, 16-22 cycles of 98°C for 15 s, 65°C for 30 s, and 72°C for 45 s, followed by 72°C for 3 min. The PCR products were loaded on a 2% E-gel (Life Technologies) for visualization and extraction, with a final buffer exchange using the PCR Micro Kit (Life Technologies). The eluted PCR DNA was used for Illumina MiSeq library preparation and 2 × 300 bp paired-end indexed sequencing at the New York Genome Center with 2–3 PCR samples multiplexed per run.

Analysis of NGS Data

The 2 × 300 bp MiSeq raw reads were assembled to single transcripts using USEARCH (Edgar and Flyvbjerg, 2015), which calculated the number of low-quality calls based on Q scores in each transcript and excluded the transcripts with more than 20 potential miscalls. Our bioinformatics pipeline, SONAR, was then used to annotate antibody transcripts (Schramm et al., 2016). Briefly, transcripts shorter than 300 nucleotides were removed. BLAST (http://www.ncbi.nlm.nih.gov/blast/) with optimized parameters was used to assign germline V, J, and C genes for each transcript with germline genes obtained from the IMGT database (http://www.imgt.org/). CDR3 was identified from boundaries of V and J genes and the conserved 2nd Cysteine in the V region and WGXG (heavy chain) or FGXG (light chain) amino acid motifs in the J region (X represents any of the 20 amino acids). The open-reading frame was then determined and the sequences other than the V(D)J region were removed. Transcripts containing frameshift or stop codons were excluded. Transcripts of heavy chains without a CH1 gene annotation were also excluded.

Antibody Lineage Analysis

For each antibody lineage of interest, the MiSeq transcripts sharing identical germline V and J genes were identified from the IgM, IgG, and IgA repertoires. Identity-divergence plot (2D plot) was employed to identify the members of each antibody lineage. Briefly, the nucleotide sequence of each transcript was aligned to the reference antibody and assigned germline V gene using MUSCLE (http://www.drive5.com/muscle), and the identity to reference antibody and the somatic hypermutation level was calculated. On the 2D plots, we selected transcripts from islands showing high identity to reference antibodies. We then analyzed the junctions (VDJ or VJ) of transcripts and removed those having junctions different from the reference antibodies. To eliminate possible PCR crossover, we removed transcripts that had a similar CDR3 sequence but did not share any somatic hypermutation in the V gene with the reference antibodies. To further select representative transcript of each lineage, we used USEARCH to cluster transcripts with 0.99 identity as described (Doria-Rose et al., 2014; Sheng et al., 2017; Wu et al., 2015). Transcripts were ranked based on sequencing depth or number of replicates, and the representative transcripts with highest coverage were selected for gene synthesis and expression. The phylogenetic analysis of the heavy chains of selected bNAb lineages was performed with the lineage members aligned using ClustalO (Sievers et al., 2011), and maximum likelihood trees were constructed using MEGA7 (Tamura et al., 2013) with GTR+G substitution model.

M1214_N1 Fab Crystal Structure Determination

The Fab fragment of M1214_N1 was produced by papain digestion as described (Burke et al., 2009; Chan et al., 2018). Preliminary crystallization condition was obtained and optimized by robotic screening using the hanging-drop vapor diffusion method. Good diffracting crystals of M1214_N1 Fab at 10 mg/mL were grown in a solution of 28% PEG8000, 1.6M calcium acetate and 20% glycerol. X-ray diffraction data were collected at beam line AMX, National Synchrotron Light Source II (NSLS II), Brookhaven National Laboratory. All datasets were processed using the XDS software package (Kabsch, 2010), and structures were determined by molecular replacement software Phaser (McCoy et al., 2007). Structural model building was carried out using COOT (Emsley and Cowtan, 2004), and refinement was calculated by Phenix (Adams et al., 2010). CαRMSDs were calculated by SuperPose (Maiti et al., 2004).

Cryo-EM sample Preparation and Data Acquisition

The CH505 SOSIP.664 trimer (Saunders et al., 2017; Zhou et al., 2017) was incubated with a 6-fold molar excess of Fab MT1214_N1 overnight at 4°C. Excess Fab was removed and the complex was concentrated to 0.2 mg/mL using a 100-kDa cutoff concentrator (Amicon Ultra, Millipore). A 4 μL aliquot of the sample was applied onto a glow-discharged C-Flat 1.2/1.3 grid (Electron Microscopy Sciences, Protochips, Inc.), blotted and then plunged into liquid ethane using FEI Vitrobot Mark IV to capture complexes in vitreous ice. Micrographs were collected on a Talos Arctica operating at 200 KeV coupled with a Gatan K2 direct electron detector using the SerialEM interface (Mastronarde, 2005). Each exposure image was collected in super-resolution mode at 36,000x magnification resulting in a pixel size of 0.58 Å/pixel, using a dose rate of ~8 e^-/pix/sec, and 250 ms exposure per frame. A total of 2,350 micrographs were collected at a random nominal defocus range between −1.5 and −3.5 μm.

Movie micrograph frames were aligned using MotionCor2 (Zheng et al., 2017) and binned by 2 to a pixel size of 1.16 Å. The contrast-transfer-function (CTF) estimation was performed for each aligned micrograph using CTFFIND4 (Rohou and Grigorieff, 2015) without dose-weighting. All single particle processing including particle picking, 2D and 3D classification was performed using RELION-3.0 (Scheres, 2012), and 3D refinement and map sharpening were carried out by cryoSPARC 2.14 (Punjani et al., 2017). Briefly, around 2,000 particles were manually picked and sorted to generate an initial model as a template for particle auto-picking. After manual inspection, a total of 209,276 particles from 783 images were extracted and sorted by two rounds of reference-free 2D class averaging along with subset selection. The sorted particles were further sorted by 3D heterogeneous refinement using an Ab-initio model and C1 symmetry. The 94,839 particles belonging to the best 3D classes were used for an unbinned refinement and reconstruction. The overall resolution of the final map was determined to 4.86 Å, based on the gold-standard Fourier shell curve using a correlation cut-off of 0.143 (Figures 5 and S2).

Model building for the CH505 SOSIP trimer in complex with Fab M1214_N1 was initiated by searching a homology model with SWISS-MODEL (Arnold et al., 2006) using CH505 SOSIP (PDB: 6UDA) as a template. Separate gp120 and gp41 models were generated, fitted into the cryo-EM map, and combined as a trimeric complex with three copies of crystal structure of Fab MT1214_N1 (PDB: 6VU2) using UCSF Chimera (Pettersen et al., 2004). Initial refinement was done using Phenix (Adams et al., 2010). Glycans were built into the map using the carbohydrate module in COOT (Emsley and Cowtan, 2004) as N-linked NAG-NAG-BMA and subsequently extended or trimmed to match EM map densities. Final refinements were performed iteratively using Phenix (Adams et al., 2010) real-space refinement with secondary structure restraint along with manual building using COOT (Emsley and Cowtan, 2004). Model validation was done using MolProbity (Chen et al., 2010). Surface contacts were calculated and colored using PyMOL (pymol.org).

QUANTIFICATION AND STATISTICAL ANALYSIS

GraphPad Prism 6.0 was used to plot the ELISA data using sigmoidal dose-response with variable slope for curve fitting and the neutralization data using 5-parameter nonlinear regression for curve fitting. No statistical analysis for group comparisons was needed in this study.

Cell Host & Microbe, Volume *27*

Supplemental Information

VSV-Displayed HIV-1 Envelope Identifies Broadly Neutralizing Antibodies Class-Switched to IgG and IgA

Manxue Jia, Rachel A. Liberatore, Yicheng Guo, Kun-Wei Chan, Ruimin Pan, Hong Lu, Eric Waltari, Eva Mittler, Kartik Chandran, Andrés Finzi, Daniel E. Kaufmann, Michael S. Seaman, David D. Ho, Lawrence Shapiro, Zizhang Sheng, Xiang-Peng Kong, Paul D. Bieniasz, and Xueling Wu

Table S1. Neutralization IC_{50} (μg/mL) of bNAbs (expressed as IgG1 or otherwise indicated) against 120 HIV-1 Env pseudoviruses. Related to Figure 1.

Virus	Clade	M4008		M1214		M1214	
		**N1**	**N1.3 dIgA2**	**N1**	**N1.2**	**N2**	**N2.2**
MS208.A1	A	>50	>50	40.2	0.090	>50	>50
Q23.17	A	0.170	0.261	1.3	0.182	>50	>50
Q461.e2	A	23.0	>50	3.0	11.4	>50	>50
Q769.d22	A	>50	>50	>50	>50	>50	>50
Q259.d2.17	A	10.8	>50	>50	>50	>50	>50
Q842.d12	A	0.082	0.414	>50	>50	0.916	>50
0260.v5.c36	A	>50	>50	>50	>50	>50	26.2
191955_A11	A (T/F)	4.0	35.1	0.205	27.1	>50	>50
191084 B7-19	A (T/F)	>50	>50	0.030	0.023	0.020	0.107
9004SS_A3_4	A (T/F)	2.0	>50	0.738	1.4	0.008	35.3
BG505	A (T/F)	0.112	0.865	>50	0.238	>50	0.518
6535.3	B	1.2	36.2	>50	>50	>50	>50
QH0692.42	B	0.203	0.096	0.045	0.035	0.111	>50
SC422661.8	B	>50	>50	0.016	0.070	0.018	0.026
PVO.4	B	0.160	0.132	0.192	2.9	0.019	0.420
TRO.11	B	0.124	7.4	>50	>50	0.027	0.017
AC10.0.29	B	1.1	>50	0.067	0.063	>50	1.3
RHPA4259.7	B	0.815	>50	0.004	0.011	0.364	>50
THRO4156.18	B	>50	>50	>50	21.1	>50	>50
REJO4541.67	B	0.133	0.152	0.015	0.145	>50	48.1
TRJO4551.58	B	>50	>50	10.2	0.025	>50	>50
WITO4160.33	B	0.197	>50	0.093	0.263	0.083	>50
CAAN5342.A2	B	>50	>50	0.024	44.3	>50	>50
JR-FL	B	0.004	0.019	0.009	0.096	0.019	0.027
Yu2	B	0.163	0.186	0.026	0.109	0.114	0.035
BL01	B	>50	>50	>50	>50	4.0	0.070
WEAU_d15_410_5017	B (T/F)	>50	>50	>50	0.336	0.059	0.041
1006_11_C3_1601	B (T/F)	0.109	>50	0.016	0.027	0.090	>50
1054_07_TC4_1499	B (T/F)	>50	>50	0.046	0.374	>50	>50
1056_10_TA11_1826	B (T/F)	0.177	0.070	0.271	1.2	>50	>50
1012_11_TC21_3257	B (T/F)	0.055	0.612	0.063	0.990	0.058	0.009
6240_08_TA5_4622	B (T/F)	13.0	>50	0.986	29.3	0.030	0.020
6244_13_B5_4576	B (T/F)	1.0	1.6	0.724	0.815	>50	0.060
62357_14_D3_4589	B (T/F)	8.6	>50	0.036	0.404	0.915	1.1
SC05_8C11_2344	B (T/F)	>50	>50	6.4	1.2	0.014	0.012
AD17	B (T/F)	0.295	0.342	0.039	0.532	0.181	>50
Du156.12	C	>50	>50	>50	>50	>50	>50
Du172.17	C	>50	>50	>50	0.468	>50	>50
Du422.1	C	>50	>50	>50	>50	>50	>50
ZM197M.PB7	C	2.6	9.2	>50	>50	>50	18.1
ZM214M.PL15	C	>50	>50	16.0	20.2	>50	>50
ZM233M.PB6	C	>50	43.5	14.2	>50	>50	>50
ZM249M.PL1	C	>50	>50	>50	>50	>50	>50
ZM53M.PB12	C	>50	>50	24.3	0.285	>50	>50
ZM109F.PB4	C	0.046	0.741	>50	>50	>50	>50
ZM135M.PL10a	C	>50	>50	>50	>50	>50	>50
CAP45.2.00.G3	C	>50	>50	>50	>50	>50	>50
CAP210.2.00.E8	C	>50	>50	>50	>50	>50	>50
HIV-001428-2.42	C	0.762	0.578	0.457	0.104	>50	>50
HIV-0013095-2.11	C	>50	>50	>50	>50	>50	>50
HIV-16055-2.3	C	>50	>50	>50	>50	>50	>50
HIV-16845-2.22	C	>50	>50	>50	>50	>50	>50
Ce1086_B2	C (T/F)	13.1	>50	2.5	11.7	>50	>50
Ce0393_C3	C (T/F)	>50	>50	>50	>50	>50	>50
Ce1176_A3	C (T/F)	>50	>50	0.463	32.3	>50	>50
Ce2010_F5	C (T/F)	9.1	>50	0.601	0.081	>50	>50
Ce0682_E4	C (T/F)	>50	27.4	>50	>50	>50	>50
Ce1172_H1	C (T/F)	17.8	12.4	>50	>50	>50	>50
Ce2060_G9	C (T/F)	>50	>50	0.019	0.084	>50	>50
Ce703010054_2A2	C (T/F)	>50	>50	>50	14.8	>50	>50
BF1266.431a	C (T/F)	1.1	1.5	>50	>50	>50	>50
246F C1G	C (T/F)	0.028	1.5	>50	0.529	>50	>50
249M B10	C (T/F)	>50	>50	>50	7.2	>50	>50
ZM247v1(Rev-)	C (T/F)	>50	>50	0.100	3.6	>50	>50
7030102001E5(Rev-)	C (T/F)	>50	>50	11.3	2.6	0.008	0.023
1394C9G1(Rev-)	C (T/F)	>50	>50	>50	>50	>50	>50
Ce704809221_1B3	C (T/F)	2.5	>50	>50	>50	>50	>50

Table S1. Neutralization IC_{50} (μg/mL) of bNAbs (expressed as IgG1 or otherwise indicated) against 120 HIV-1 Env pseudoviruses. Related to Figure 1. ***Continued.***

**Virus**	**Clade**	**M4008**		**M1214**		**M1214**	
		**N1**	**N1.3 dIgA2**	**N1**	**N1.2**	**N2**	**N2.2**
CNE19	BC	0.269	0.156	0.008	0.024	>50	>50
CNE20	BC	>50	>50	3.3	>50	>50	>50
CNE21	BC	>50	>50	0.038	47.5	>50	>50
CNE17	BC	>50	13.1	0.075	0.238	>50	>50
CNE30	BC	>50	>50	0.963	2.8	>50	>50
CNE52	BC	>50	>50	>50	>50	>50	>50
CNE53	BC	>50	>50	22.8	3.6	>50	>50
CNE58	BC	22.2	0.284	>50	45.3	>50	>50
620345.c01	CRF01_AE	>50	>50	>50	>50	>50	>50
C1080.c03	CRF01_AE	>50	>50	>50	>50	>50	>50
R2184.c04	CRF01_AE	>50	>50	0.008	0.099	>50	>50
R1166.c01	CRF01_AE	>50	>50	7.9	>50	>50	>50
R3265.c06	CRF01_AE	>50	>50	0.230	>50	>50	>50
C2101.c01	CRF01_AE	>50	>50	0.006	0.123	>50	>50
C3347.c11	CRF01_AE	>50	>50	0.517	>50	>50	>50
C4118.c09	CRF01_AE	>50	>50	6.9	30.0	>50	>50
CNE5	CRF01_AE	>50	>50	0.973	0.255	>50	>50
BJOX009000.02.4	CRF01_AE	>50	>50	13.6	3.3	>50	>50
BJOX015000.11.5	CRF01_AE (T/F)	>50	>50	0.056	0.323	>50	>50
BJOX010000.06.2	CRF01_AE (T/F)	>50	>50	0.023	0.072	>50	>50
BJOX025000.01.1	CRF01_AE (T/F)	>50	>50	0.008	0.098	>50	>50
BJOX028000.10.3	CRF01_AE (T/F)	>50	>50	0.040	>50	>50	>50
T257-31	CRF02_AG	>50	>50	4.6	>50	>50	>50
928-28	CRF02_AG	>50	>50	>50	>50	>50	>50
263-8	CRF02_AG	>50	>50	>50	>50	0.770	>50
T250-4	CRF02_AG	>50	>50	>50	>50	0.042	>50
T251-18	CRF02_AG	>50	>50	>50	>50	>50	>50
T278-50	CRF02_AG	>50	>50	>50	>50	11.1	>50
T255-34	CRF02_AG	>50	>50	>50	>50	>50	>50
211-9	CRF02_AG	>50	>50	>50	>50	0.057	0.017
235-47	CRF02_AG	>50	>50	0.734	2.6	16.4	>50
X1193_c1	G	>50	>50	0.018	0.026	>50	>50
P0402_c2_11	G	12.7	>50	>50	>50	>50	>50
X1254_c3	G	0.926	>50	0.046	11.4	>50	>50
X2088_c9	G	>50	>50	>50	>50	>50	>50
X2131_C1_B5	G	>50	30.9	>50	>50	>50	>50
P1981_C5_3	G	>50	>50	1.1	17.2	>50	>50
X1632_S2_B10	G	0.077	>50	0.044	0.822	>50	>50
3016.v5.c45	D	>50	>50	>50	20.0	>50	>50
A07412M1.vrc12	D	>50	>50	0.115	1.6	>50	>50
231965.c01	D	>50	>50	2.3	0.153	0.054	0.768
231966.c02	D	>50	>50	0.027	0.068	>50	>50
3817.v2.c59	CD	>50	>50	13.4	>50	0.290	0.181
6480.v4.c25	CD	1.5	>50	0.067	0.178	0.130	0.068
6952.v1.c20	CD	>50	>50	>50	>50	>50	>50
6811.v7.c18	CD	0.896	0.528	0.007	0.120	>50	>50
89-F1_2_25	CD	>50	>50	>50	>50	>50	>50
3301.v1.c24	AC	45.3	>50	>50	0.391	>50	>50
6041.v3.c23	AC	0.261	0.424	>50	>50	>50	>50
6540.v4.c1	AC	0.860	6.7	>50	>50	36.2	>50
6545.v4.c1	AC	0.730	12.7	0.043	>50	6.7	>50
0815.v3.c3	ACD	0.486	0.377	>50	>50	0.055	>50
3103.v3.c10	ACD	>50	33.7	10.0	0.573	>50	>50
**MAb breadth (%)**	**N=120**	**45 (38%)**	**33 (28%)**	**68 (57%)**	**69 (57%)**	**31 (26%)**	**24 (20%)**
**Clone breadth (%)**		**50 (42%)**		**78 (65%)**		**37 (31%)**	
**MAb breadth (%) IC_{50} < 1 μg/mL**		**26 (22%)**	**18 (15%)**	**48 (40%)**	**42 (35%)**	**26 (22%)**	**18 (15%)**
**Clone breadth (%) IC_{50} < 1 μg/mL**		**27 (23%)**		**59 (49%)**		**29 (24%)**	
**MAb geometric mean (μg/mL)**		**0.755**	**1.408**	**0.255**	**0.702**	**0.154**	**0.199**
**Clone geometric mean**		**0.766**		**0.188**		**0.159**	

**IC_{50} color code**	< 1	1.0 - 50	>50 (μg/mL)

Table S2. Key contacts of the M1214_N1 epitope on gp120 and their levels of conservation among 5,164 global Env sequences (version year 2016) retrieved from the Los Alamos HIV sequence database (http://www.hiv.lanl.gov/). Related to Figure 5.

Gp120 residues	Gp120 regions	Buried surface ($Å^2$)#	Level of conservation*
Q183	V2	14.9	0.572
D185	V2	34.1	0.365
I194	V2	15.2	0.806
N195	V2	11.3	0.670
N280	C2, loop D	23.0	0.953
S365	C3, CD4 binding loop	18.7	0.787
G366	C3, CD4 binding loop	10.4	0.990
D368	C3, CD4 binding loop	25.9	0.992
L369	C3, CD4 binding loop	15.1	0.639
E370	C3, CD4 binding loop	32.8	0.987
Y384	C3, CD4 binding site	25.7	0.968
K421	C4	15.9	0.892
I424	C4	16.5	0.797
N425	C4	39.1	0.871
D457	C4	22.0	0.981
R469	V5	23.0	0.981
Geometric Mean			0.804

#The buried surface areas were calculated using ICM software package (https://www.molsoft.com/), and only residues with surface contact areas > 10 $Å^2$ are listed.
*Level of conservation was calculated using an entropy scoring method implemented in the R package bio3d.

Table S3. M1214_N1 Fab crystallographic data collection and refinement statistics. Related to Figure 5.

Protein	M1214_N1 Fab
**PDB ID**	6VU2
**Data collection***	
Space group	P21
Cell dimensions	
a, *b*, *c* (Å)	50.7, 66.9, 70.2
α, β, γ (°)	90.0, 108.7, 90.0
Resolution (Å)	27.4-2.2 (2.27-2.19)
R_{sym} or R_{merge}	5.9 (40.7)
$I/\delta I$	10.6 (1.9)
$CC_{1/2}$	0.996 (0.933)
Completeness (%)	95.2 (72.0)
Redundancy	2.4 (1.8)
**Refinement**	
Resolution (Å)	29.4-2.2
No. reflections	37,801
R_{work}/R_{free}	17.7/21.6
No. atoms	
Protein	3258
Water	187
Wilson B-factor	37.0
r.m.s. deviations	
Bond lengths (Å)	0.007
Bond angles (º)	0.87
Ramachandran statistics	
Favored (%)	97.2
Outliers (%)	0.47

*Values in parenthesis denote highest resolution shell.

Table S4. Cryo-EM data collection, processing and refinement statistics. Related to Figure 5.

M1214_N1 Fab/CH505 chimeric SOSIP.664 (EMD-21456, PDB 6VY2)	
**Data collection and processing**	
Microscope	FEI Talos Arctica
Camera	Gatan K2 Summit
Magnification	36,000
Voltage (kV)	200
Electron exposure (e^{-}/Å^2)	80
Defocus range (μm)	-1.5 to -3.5
Pixel size (Å)	1.16
Initial particle (no.)	209,276
Final particle (no.)	94,839
Symmetry imposed	C3
Map resolution (masked, Å)	4.86
FSC threshold	0.143
Map sharpening *B* factor (Å^2)	-201.5
**Refinement**	
Initial models used (PDB ID)	6UDA, 6VU2
Model composition	
Non-hydrogen atoms (no.)	21429
Protein residues (no.)	2442
Sugar residues (no.)	180
RMSD	
Bond lengths (Å)	0.006
Bond angles (°)	0.905
Validation	
Clashscore	18.0
Poor rotamers (%)	5.0
Ramachandran statistics (%)	
Favored	93.8
Allowed	5.7
Outliers	0.5

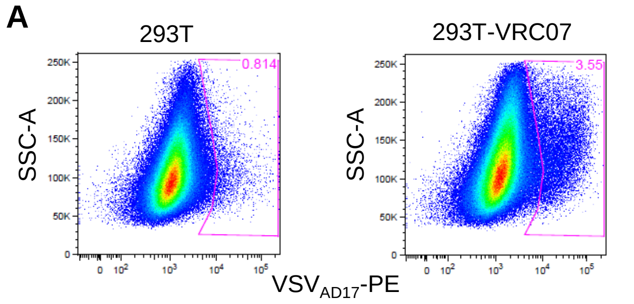

B

Virus (clade)	Plasma Neutralization ID_{50} (reciprocal dilution)	
	M4008	M1214
Q23.17 (A)	248	2,488
Q842.d12 (A)	2,080	1,440
BG505 (A)	124	994
AD17 (B)	410	256
JR-FL (B)	2,996	8,137
Yu2 (B)	429	682
AC10.29 (B)	338	305
BL01 (B)	86	120
Du156.12 (C)	176	242
ZM109.4 (C)	111	<50

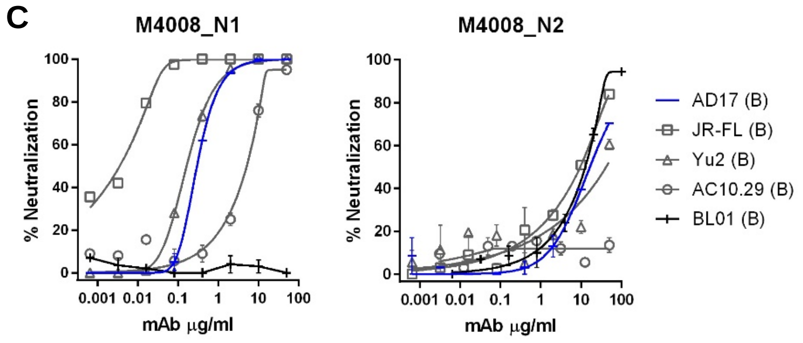

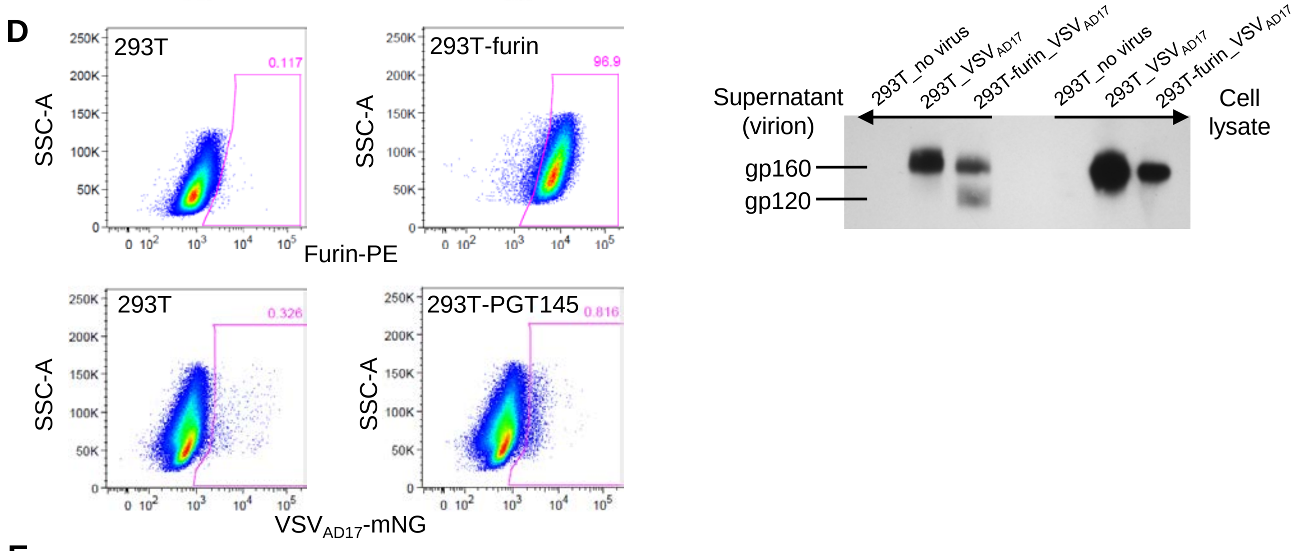

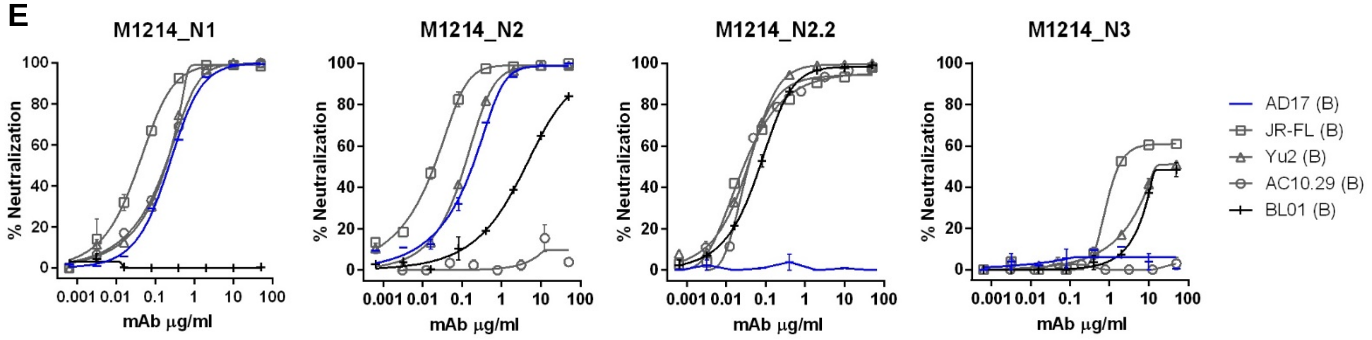

Figure S1. Evaluation of VSV$_{AD17}$ as B cell probe for HIV-1 bNAb isolation. Related to Figure 1.
(**A**) Flow cytometry analysis of VSV$_{AD17}$-PE binding to 293T cells and to 293T cells expressing membrane-bound VRC07 (293T-VRC07). SSC-A: side-scatter area.
(**B**) Neutralization ID_{50}, shown as plasma reciprocal dilution, of two donors from the Montreal HIV-1 infection cohort, against 10 HIV-1 Env strains as indicated, with the probe strain AD17 in blue.
(**C**) Neutralization screening of bNAbs from donor M4008 against five clade B viruses as indicated.
(**D**) Flow cytometry analysis of furin expression in 293T cells with and without stable transduction of the human furin gene; Western blot with HIVIG showing the uncleaved gp160 and cleaved gp120 in virion and in cell lysate from 293T versus 293T-furin; flow cytometry analysis of VSV$_{AD17}$-mNG staining of 293T cells and 293T cells expressing membrane-bound PGT145 (293T-PGT145).
(**E**) Neutralization screening of bNAbs from donor M1214 against five clade B viruses as indicated, with the probe strain AD17 in blue.

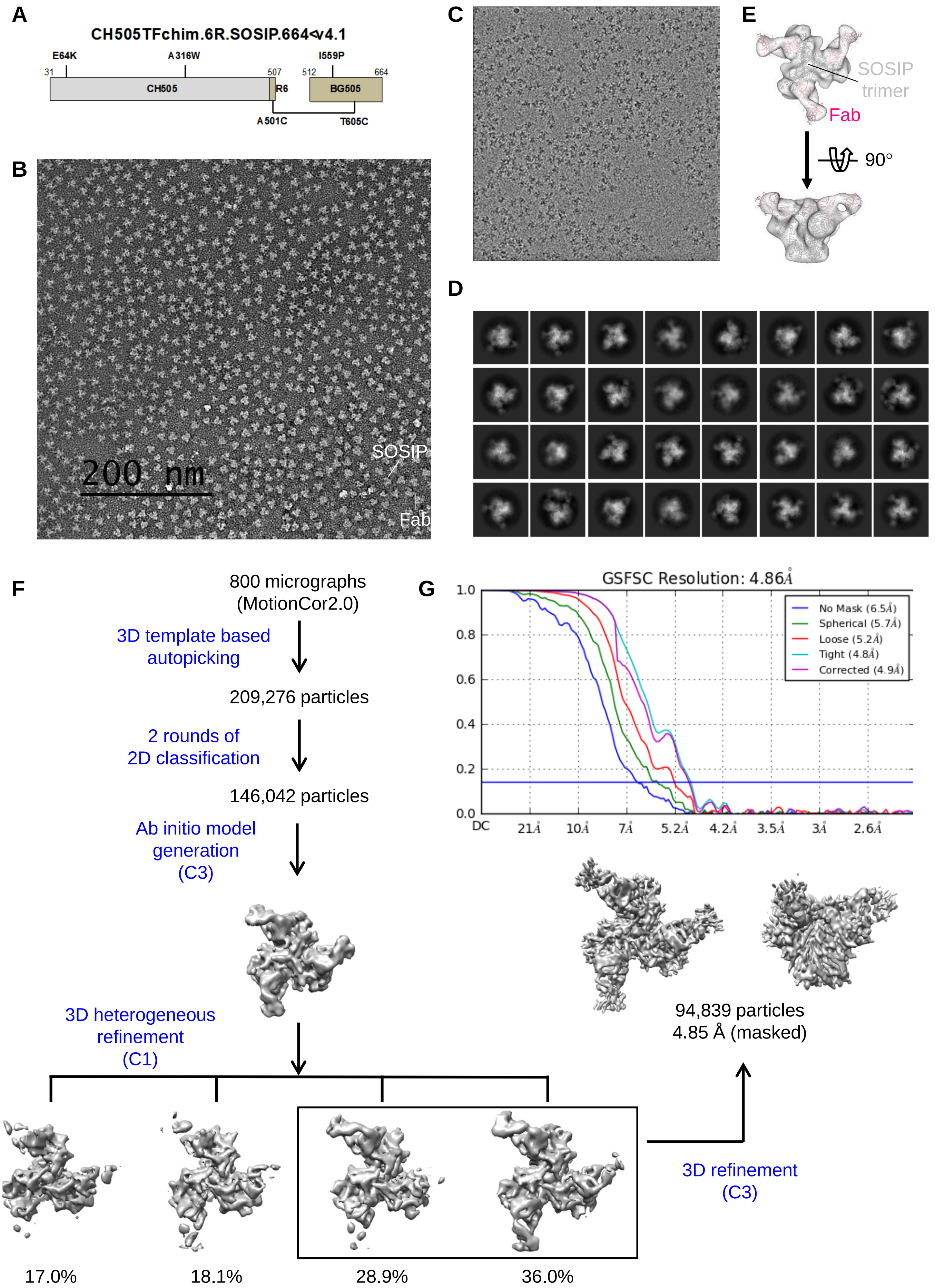
A
CH505TFchim.6R.SOSIP.664v4.1
E64K
A316W
I559P
31
507
512
664
CH505
R6
BG505
A501C
T605C
B
200 nm
SOSIP
Fab
C
D
E
SOSIP
trimer
Fab
90°
F
800 micrographs
(MotionCor2.0)
3D template based
autopicking
209,276 particles
2 rounds of
2D classification
146,042 particles
Ab initio model
generation
(C3)
3D heterogeneous
refinement
(C1)
17.0%
18.1%
28.9%
36.0%
3D refinement
(C3)
94,839 particles
4.85 Å (masked)
G
GSFSC Resolution: 4.86Å
No Mask (6.5Å)
Spherical (5.7Å)
Loose (5.2Å)
Tight (4.8Å)
Corrected (4.9Å)
1.0
0.8
0.6
0.4
0.2
0.0
DC
21Å
10Å
7Å
5.2Å
4.2Å
3.5Å
3Å
2.6Å

Figure S2. Cryo-EM sample preparation, data collection and processing. Related to Figure 5.
(A) Schematic construct of the chimeric CH505 SOSIP, in which the sequence of gp41 along with the C-terminal portion of gp120 was replaced by that of BG505.
(**B**) A representative micrograph of the negative staining of M1214_N1 Fab/CH505 SOSIP complex, showing the homogeneity of the complex sample, in which more than 90% of the SOSIP trimer was bound with three Fabs (inset) estimated by 2D class averaging.
(**C**) A representative cryo-EM micrograph of M1214_N1 Fab/CH505 SOSIP complex.
(**D**) 2D averaging of cryo-EM particle images shows the structural features for both SOSIP trimer and Fab.
(**E**) 3D initial model of the M1214_N1 Fab/CH505 SOSIP complex, fitted with a CH505 SOSIP trimer (PDB 6UDA) and three crystallographic M1214_N1 Fabs (PDB 6VU2).
(**F**) Workflow of data processing performed using cryoSPAC.
(**G**) FSC curve for 3D reconstruction of the cryo-EM map. The average resolution is estimated to be 4.86 Å on the basis of the FSC value of 0.143.

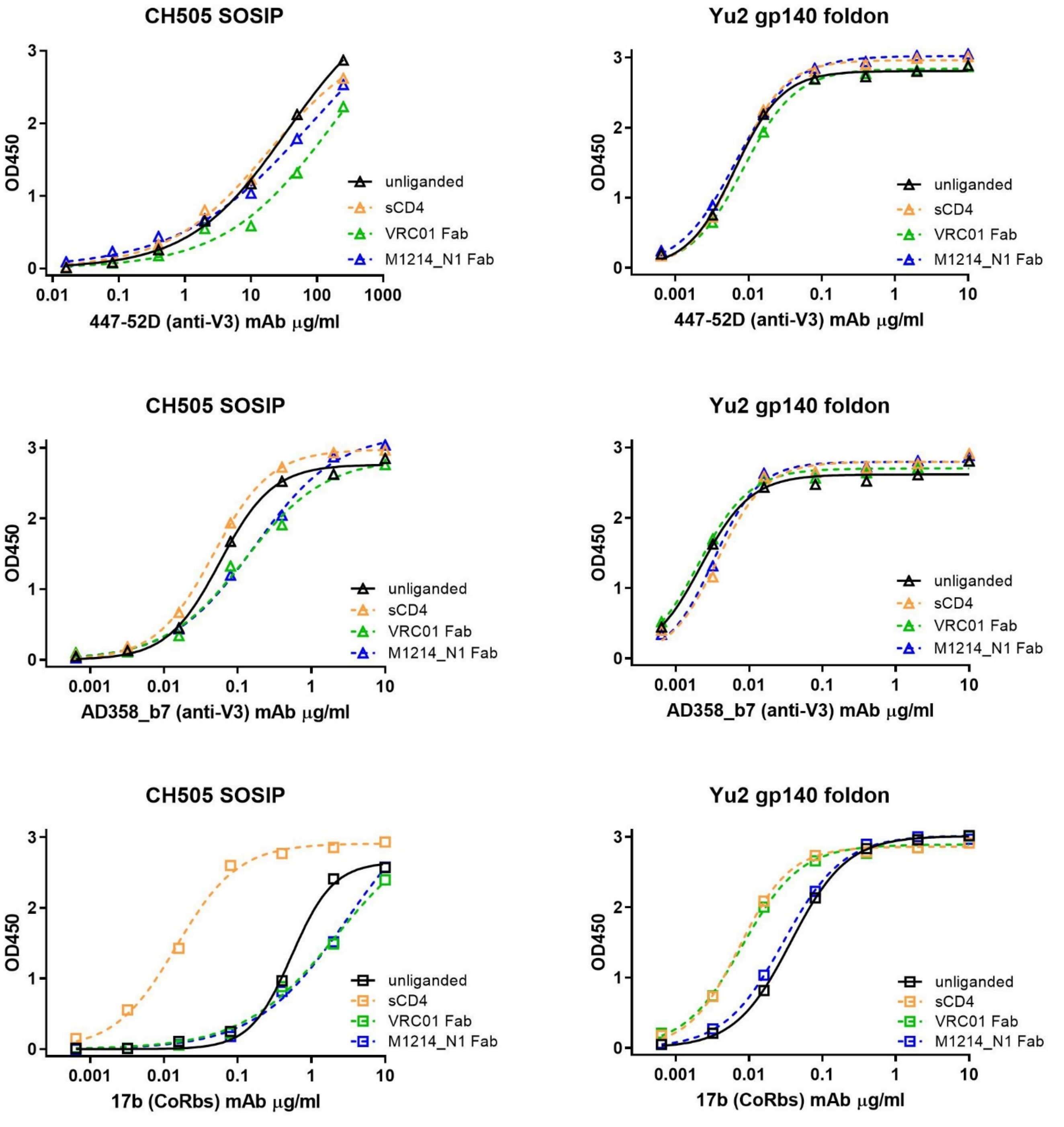

Figure S3. Assessment of the conformational change of CH505 SOSIP upon M1214_N1 Fab binding. Related to Figure 5.

The conformational change of CH505 SOSIP (left) upon 2-domain sCD4, VRC01 Fab, and M1214_N1 Fab binding was each assessed by ELISA with the anti-V3 crown mAbs 447-52D (top) and AD358_b7 (middle), as well as the coreceptor-binding site (CoRbs) mAb 17b (bottom), all of which preferentially recognize the CD4-induced "open" Env conformation. Yu2 gp140 foldon (right) was included as a control for gp140 that adopts an "open" Env conformation.
